# Transcriptional activation of endogenous Oct4 via the CRISPR/dCas9 activator ameliorates Hutchinson‐Gilford progeria syndrome in mice

**DOI:** 10.1111/acel.13825

**Published:** 2023-03-25

**Authors:** Junyeop Kim, Yerim Hwang, Sumin Kim, Yujung Chang, Yunkyung Kim, Youngeun Kwon, Jongpil Kim

**Affiliations:** ^1^ Laboratory of Stem Cells & Cell reprogramming, Department of Chemistry Dongguk University 100‐715 Seoul Korea; ^2^ Laboratory of Protein Engineering, Department of Biomedical Engineering Dongguk University 04620 Seoul Korea

**Keywords:** aging, CRISPR/dCas9, Hutchinson‐Gilford progeria syndrome, Oct4, rejuvenation

## Abstract

Partial cellular reprogramming via transient expression of Oct4, Sox2, Klf4, and c‐Myc induces rejuvenation and reduces aged‐cell phenotypes. In this study, we found that transcriptional activation of the endogenous Oct4 gene by using the CRISPR/dCas9 activator system can efficiently ameliorate hallmarks of aging in a mouse model of Hutchinson‐Gilford progeria syndrome (HGPS). We observed that the dCas9‐Oct4 activator induced epigenetic remodeling, as evidenced by increased H3K9me3 and decreased H4K20me3 levels, without tumorization. Moreover, the progerin accumulation in HGPS aorta was significantly suppressed by the dCas9 activator‐mediated Oct4 induction. Importantly, CRISPR/dCas9‐activated Oct4 expression rescued the HGPS‐associated vascular pathological features and lifespan shortening in the mouse model. These results suggest that partial rejuvenation via CRISPR/dCas9‐mediated Oct4 activation can be used as a novel strategy in treating geriatric diseases.

## Graphical abstract

In this study, we found that transcriptional activation of the endogenous Oct4 gene by using the CRISPR/dCas9 activator system can efficiently induce partial reprogramming via resetting of aging‐related epigenetic modifications. Also, CRISPR/dCas9 activator‐mediated temporal activation of endogenous Oct4 can efficiently rescue the HGPS‐associated vascular pathological features and lifespan shortening in the mouse model.

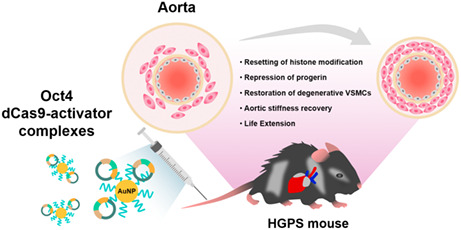

**Abbreviations**
AuNP: gold nanoparticle
dCas9: a mutant Cas9 termed dead Cas9
mutLMNA: G608G laminA/C mutation
PEI: polyethylenimine
SAM: synergistic activation mediator
sgRNA: single‐guide RNA
VSMC: vascular smooth muscle cell

## 1 INTRODUCTION

Terminally differentiated somatic cells can be reprogramed into pluripotent stem cells (induced pluripotent stem cells, iPSCs) via overexpression of the four Yamanaka factors Oct3/4, Sox2, Klf4, and c‐Myc (OSKM) (Takahashi & Yamanaka, 2006). For decades, numerous studies have demonstrated that cell fate can be altered by the forced expression of transcription factors and thereby opened exciting avenues for the potential use of cell‐reprogramming approaches for the treatment of many diseases. Particularly, these studies raised the feasibility of partial reprogramming toward pluripotency as a new anti‐aging strategy (Simpson et al., 2021; Taguchi & Yamada, 2017; Zhang et al., 2020). For example, cyclic expression of OSKM has been shown to improve age‐associated cellular phenotypes in progeria mice (Ocampo et al., 2016). Moreover, several groups have observed that partial in vivo reprogramming enhances the regenerative capacity of the muscle (Wang et al., 2021), heart (Chen et al., 2021) and repairs traumatic brain injury in old mice (Seo et al., 2016). Additionally, partial induction of OSKM or OKS improves cognitive functions and ameliorates aging‐associated features in brain cells and retinal ganglion cells in mice (Xiao et al., 2021). Also, local expression of OSKM in the skin improves wound healing by reducing the transdifferentiation into myofibroblasts (Doeser et al., 2018; Kurita et al., 2018). Thus, these studies indicate that transient expression of OSKM can erase certain cellular marks of aging and increase regenerative capacity. However, most of these studies were based on the overexpression of OSKM for rejuvenation reprogramming, and these approaches have the risk of inducing teratoma formation during the process of de‐differentiation (Abad et al., 2013).

Particularly, previous studies have indicated that endogenous Oct4 expression is critical in inducing pluripotency, and reactivation of endogenous Oct4 plays an important role in the epigenetic reprogramming process to remove the original somatic‐cell epigenetic landscape and establish the iPSC‐specific epigenetic landscape (Kang et al., 2009; Pan et al., 2002; Shi & Jin, 2010; Wu & Scholer, 2014). For example, activation of the endogenous Oct4 gene by using small‐molecule compounds (An et al., 2019; Heng et al., 2010; Hou et al., 2013) or Tet1‐mediated demethylation enables de‐differentiation for the cellular reprogramming and establishment of the pluripotency state without exogenous Oct4 expression (Gao et al., 2013). Notably, induction of the endogenous pluripotent network by Oct4, which maintains pluripotency, is also critical for initiating reprogramming (Buganim et al., 2012; Kim et al., 2021; Pardo et al., 2010; Shu et al., 2013; Velychko et al., 2019). Collectively, these results have suggested that activation of endogenous Oct4 transcription is necessary to initiate epigenetic remodeling and transcriptome resetting to reprogram somatic cells into iPSCs. From this point of view, induction of endogenous Oct4 expression can be envisioned to trigger rejuvenation reprogramming.

Recently, the CRISPR/Cas9 system has been modified to control gene expression. In this modified system, a mutant Cas9 termed dead Cas9 (dCas9), which can perform RNA‐dependent DNA‐binding without the endonuclease activity (Brezgin et al., 2019; Qi et al., 2013) is fused to a functional domain, such as synergistic activation mediator (SAM), VPR, or KRAB, to activate or repress endogenous gene expression by binding to a single‐guide RNA (sgRNA) (Perez‐Pinera et al., 2013). In particular, the dCas9‐mediated transcriptional activator (CRISPRa) has been successfully used to efficiently activate endogenous genes in the process of cellular reprogramming (Liu et al., 2018), neurons (Russo et al., 2021), and cardiac cells (Kwon et al., 2020). Moreover, recent studies have shown the therapeutic use of the CRISPR/dCas9 activation system in neurodegenerative diseases, metabolic and inflammatory diseases, cancer, and several other genetic disorders, suggesting that this system can be used to treat diseases (Park et al., 2021; Park & Kim, 2022). Notably, since this system is not accompanied by irreversible changes in the DNA sequence, it can enable efficient and precise induction of partial reprogramming to sufficiently alleviate the cellular phenotypes of aging without side effects.

In this study, we examined whether dCas9‐mediated transcriptional activation of Oct4 could suppress the premature aging caused by a mutation in the LMNA gene resulting in truncated progerin protein, in mice in vivo and in vitro (Cao et al., 2007; Hennekam, 2006). We first confirmed that endogenous Oct4 activation via the CRISPR activation system efficiently triggers partial reprogramming for rejuvenation. We next showed the number of epigenetic changes in response to endogenous Oct4 expression. Aging‐associated changes in the levels of heterochromatin marks, such as dysregulation of H3K9me3 and H4K20me3 levels, were alleviated by the dCas9‐Oct4 activator (Benayoun et al., 2015; Liu et al., 2013; Ocampo et al., 2016). Furthermore, we observed reduced aging‐associated cellular defects, such as premature cellular senescence, DNA damage, clustering of nuclear pores, and delayed cell proliferation, by the endogenous Oct4 activation. Most prominently, we showed that the rejuvenation reprogramming induced by endogenous Oct4 expression effectively suppressed the age‐related phenotypes in a mouse model of Hutchinson‐Gilford progeria syndrome (HGPS). Thus, our data indicate that temporal induction of endogenous Oct4 expression by using the CRISPR/Cas9 Oct4 activator may be an effective rejuvenation strategy to address aging‐related phenotypes and diseases.

## 2 MATERIALS AND METHODS

### 2.1 Preparation of the dCas9‐activator

To produce the dCas9 activator vector, dCas9 activator vectors were prepared using PB‐UniSAM (Addgene, #99866) vector. The PB‐uniSAM vector was linearized via BbsI digestion at 37 °C for 2 h. Two single‐stranded oligonucleotides (Table S1, Oct4 sgRNA #1–10) are then annealed in a 10 μL reaction volume by using T4 PNK (NEB) with 3 μL of each oligo. Fifty nanograms of purified BbsI‐digested plasmid was ligated to 7 μL of annealed sgRNA ligation buffer. DH5α competent E. coli was used for transformation of the ligated vector, which was subsequently amplified using these cells, purified, and then confirmed via Sanger sequencing.

### 2.2 Gene delivery

Conjugation of Polyethylenimine (PEI) with AuNPs modified with six different thiol ligands (RRRGYC) was used for gene delivery (Chang et al., 2019). AuNPs/dCas9‐activator/PEI nano complexes (5 μg ligand‐modified AuNPs, 1 μg dCas9‐activator, and 3 μg Polyethylenimine) were prepared in a total volume of 50 μL. Afterward, mouse tail‐tip fibroblasts or NIH/3T3 cells were incubated with the AuNPs/dCas9‐activator/PEI complexes for 4 h at 37°C, and then, the medium was replaced with DMEM containing 10% FBS.

### 2.3 Animal experiments

All procedures involving animals used in experiments were performed in accordance with institutional guidelines for animal use and received ethical approval from the Institutional Animal Care and Use Committee at Dongguk University (IACUC‐2019‐043‐2). Control wild‐type C57BL/6 male mice (Korea Research Institute of Bioscience & Biotechnology) and the HGPS mice carrying the G608G laminA/C mutation (The Jackson Laboratory, stock number 101667) were used. All the mice were maintained on a 12 h/12 h light/dark cycle (light on and off at 9:00 and 21:00, respectively) at 23 ± 1°C with free access to food and water. For in vivo delivery of Cas9 activator, AuNPs/PEI nanocomplexes containing dCas9‐activator were prepared as previously described (Chang et al., 2019). AuNPs/dCas9 activator nanocomplexes diluted with 0.9% saline and injected into the tail vein of five‐month‐old control and HGPS male mice. All mice were anesthetized with tribromoethanol (Avertin, 120 mg/kg) for the tail‐vein injection. After injection, the mice were kept warm until full recovery from anesthesia. Two weeks after first injection, the same mice were subject to the second injection of AuNPs/dCas9 activator nanocomplexes. Seven weeks after the second injection, these mice were subjected to biochemical and histological analyses.

### 2.4 Assessment of off‐target modification

Potential off‐target sites were predicted using Cas‐OFFinder (http://www.rgenome.net/cas‐offinder/), which searches for CRISPR/Cas‐derived RNA‐guided Endonucleases (RGENs) off target sites. Briefly, considering the degeneracy in PAM recognition, Cas‐OFFinder complies with all 23 bp DNA sequences consisting of 20 bp sequences corresponding to sgRNA sequence of on‐target and the 5′‐NRG‐3′ PAM sequences. All compiled sequences are then compared to the on‐target sequence, and the number of mismatched bases in the 20‐bp sgRNA sequence is counted. The top 10 potential sgRNA binding off‐target sites were selected considering the diversity of 23 homologous chromosomes among those with 2–4 mismatches compared to the on‐target sequence, except for off‐target sites located in introns. There are no off‐target sites less than 1 mismatch. (PAM type: 5′‐NGG‐3′, Mismatch number: less than 4, Target Genome: Mus musculus (mm10)–Mouse).

### 2.5 Cell culture

DMEM (Gibco) supplemented with 1% penicillin/streptomycin (P/S, Gibco) and 10% fetal bovine serum (FBS, Gibco). All cells were incubated in a humidified atmosphere of 5% CO_2_ at 37°C. Every 3 months, cells were certified using the short‐tandem repeat assay provided by KogeneBiotech and confirmed to be negative for mycoplasma contamination by using the MycoSensor PCR Assay Kit (Agilent). No replicate was excluded from the data analysis. Tail‐tip fibroblasts were isolated from a transgenic mouse that expresses eGFP under the control of the mouse Oct4 locus (Oct4‐eGFP mouse). A 1–2 mm piece of the tail tip was removed from each mouse and rinsed well with 70% ethanol for 30 s. Tissues were washed with fresh phosphate‐buffered saline (PBS), minced well on a 10 cm tissue culture dish by using a sterile razor blade, and then incubated with EDTA‐containing 0.25% trypsin (Gibco) for 30 min at 37°C. Tail‐tip fibroblasts were cultured until confluence.

### 2.6 RNA isolation and reverse transcription quantitative polymerase chain reaction (RT‐qPCR)

All RNA was collected and purified using RNA preparation kit (Philekorea, #EP52200) by following the manufacturer's instructions. RNA was then reverse‐transcribed to cDNA by using AccuPower® CycleScript RT PreMix (Bioneer, #K‐2044‐B). RT‐qPCR analysis was performed using AccuPower® PCR PreMix (Bioneer, #K‐2016) with suitable primers. All the reactions were performed using a Rotor‐Gene Q real‐time PCR cycler (Qiagen).

### 2.7 Western blotting

Cells and tissues were lysed in RIPA buffer with a protease inhibitor cocktail. Protein concentration was evaluated using Quick Start Bradford protein assay (Bio‐Rad). SDS‐PAGE and Western blot analysis were performed in accordance with standard procedures, and the protein bands of interest were detected using an ECL detection kit. The antibodies used were anti‐H3K9me3 (Abcam, #ab8898), anti‐H4K20me3 (Abcam, #ab78517), anti‐Oct4 (Abcam, #ab18976, santacruz, #sc‐5279), anti‐beta actin (abfrontier, #LF‐PA0207), and anti‐H3 (Abcam, #ab6002) antibodies.

### 2.8 Immunofluorescence staining

Cells were cultured in 24‐well plates for 24h following transfection with the dCas9/Oct4 activator. For immunofluorescence staining, attached cells were washed with 1X PBS and then fixed with 4% paraformaldehyde (PFA) at room temperature for 10 min. Afterward, they were washed twice with PBS and then blocked for 3 h at room temperature with 1X PBS containing 3% bovine serum albumin (BSA) and 0.1% Triton X‐100. Primary antibodies were used at the dilution recommended by the manufacturer. The cells were stained with anti‐Oct4 (abcam, #ab18976, santacruz, #sc‐5279), rabbit anti‐phospho‐gammaH2AX (Cell signaling, #9718), rabbit anti‐cleaved caspase‐3 (Cell signaling, #9661), or α‐SMA (Invitrogen, # 14–9760‐82) for 24h at 4°C and then incubated with appropriate secondary antibodies conjugated to Alexa488 or Alexa 594 (Invitrogen #A11001, #A11005, #A11034, #A11012), followed by DAPI staining. The number of cells in 10 randomly selected fields on the plate was calculated using a versatile confocal microscope (LSM‐800, Zeiss) at 200× magnification, and three independent experiments were performed.

### 2.9 Virus generation

Lentiviral vectors that contained mouse complementary DNAs (cDNAs) for FUW‐TetO‐mutant laminA/C (Progerin) were generated according to the standard procedures. Lentiviruses were produced in HEK293T cells grown in DMEM containing 10% FBS, 1% P/S, and 1% Geneticin. The day before transfection, 2 × 10^7^ cells were seeded on a 15 cm culture dish containing the growth medium. The next day, the cells were co‐transfected with the lentivirus constructs psPAX2, pMD2.G, and FUW‐M2rtTA via calcium‐phosphate co‐precipitation. The lentivirus constructs for psPAX2, pMD2.G, and FUW‐M2rtTA were purchased from Addgene (#12260, #12259 and #20342). For the FUW‐tetO‐Progerin construct, Progerin (mutant LamicA/C) cDNA was amplified from cDNA library derived from HGPS patient fibroblasts (Coriell institute, #AG11498) and cloned into pCW57.1 (Addgene, #41393). The mutant LMNA (Progerin) sequence was confirmed by Sanger sequencing. The culture medium was replaced 24 h after the transfection, and the viruses were harvested 72 h after transfection. Supernatants containing the lentivirus were collected, centrifuged to eliminate cell debris, and filtered through a 0.45 μm filter. Viruses were pelleted via standard ultracentrifugation. The pellet was resuspended with cold PBS. Before the viral titers (infectious unit mL − 1) were determined, FUW‐GFP was used to monitor GFP expression as a control for calculate the multiplicity of infection.

### 2.10 Plasmid construction and transfection

The expression constructs that contained the dCas9/Oct4 activator were subcloned from each FUW‐tetO vector described previously. For the transfection, the fibroblasts were seeded at 75%–80% confluency 24 h before transduction. Three consecutive transfections were performed for 36 h by using the Lipofectamine3000 reagent (Thermo Fisher). A monolayer of fibroblasts in a 6‐well culture dish was transfected with the constructs at a 5:2 or 6:2 ratios of Lipofectamine3000 Reagent to DNA and then incubated for 12‐h according to the manufacturer's protocol. The culture medium was changed 12‐h after the transfection.

### 2.11 FACS analysis

Cells were dissociated into single cells by incubating them in 0.25% trypsin in EDTA at 37°C for 3 min and then rinsed twice with 1X PBS to wash away the remaining trypsin. They were then fixed with 4% paraformaldehyde (PFA) for 15 min. After the cells were washed with 1X PBS, they were resuspened in the flow cytometry buffer (PBS containing 1% BSA). The cells were analyzed using a C6 flow cytometer (BD Biosiences Franklin, NJ, USA) wiht FlowJo software (Tree Star, Inc.).

### 2.12 Senescence‐associated beta‐galactosidase assay

Cells were fixed in 4% paraformaldehyde (PFA) for 5 min at room temperature. Next, they were washed twice with PBS and then incubated overnight at 37°C in a staining solution containing 5 mM K4 (Fe[CN]6) 3H_2_O, 40 mM citric acid/Na phosphate buffer, 2 mM magnesium chloride, 5 mM K3 (Fe[CN]6), 150 mM sodium chloride, and 1 mg/mL X‐gal. Cells were then washed twice with PBS and once with methanol. The cells were dried and then photographed using bright‐field microscopy.

### 2.13 Statistical analysis

All the data are presented as the mean ± SEM from three independent experiments. Differences were considered significant at *p* < 0.05 (*p < 0.05, **p < 0.005). The significance of intergroup differences was assessed via one‐way or two‐way ANOVA followed by Tukey's multiple comparison test and Student's *t* test for two‐component comparisons after a normal distribution was confirmed.

## 3 RESULTS

### 3.1 Transcriptional activation of the endogenous Oct4 gene via the CRISPR/dCas9 activator system

**FIGURE 1:**
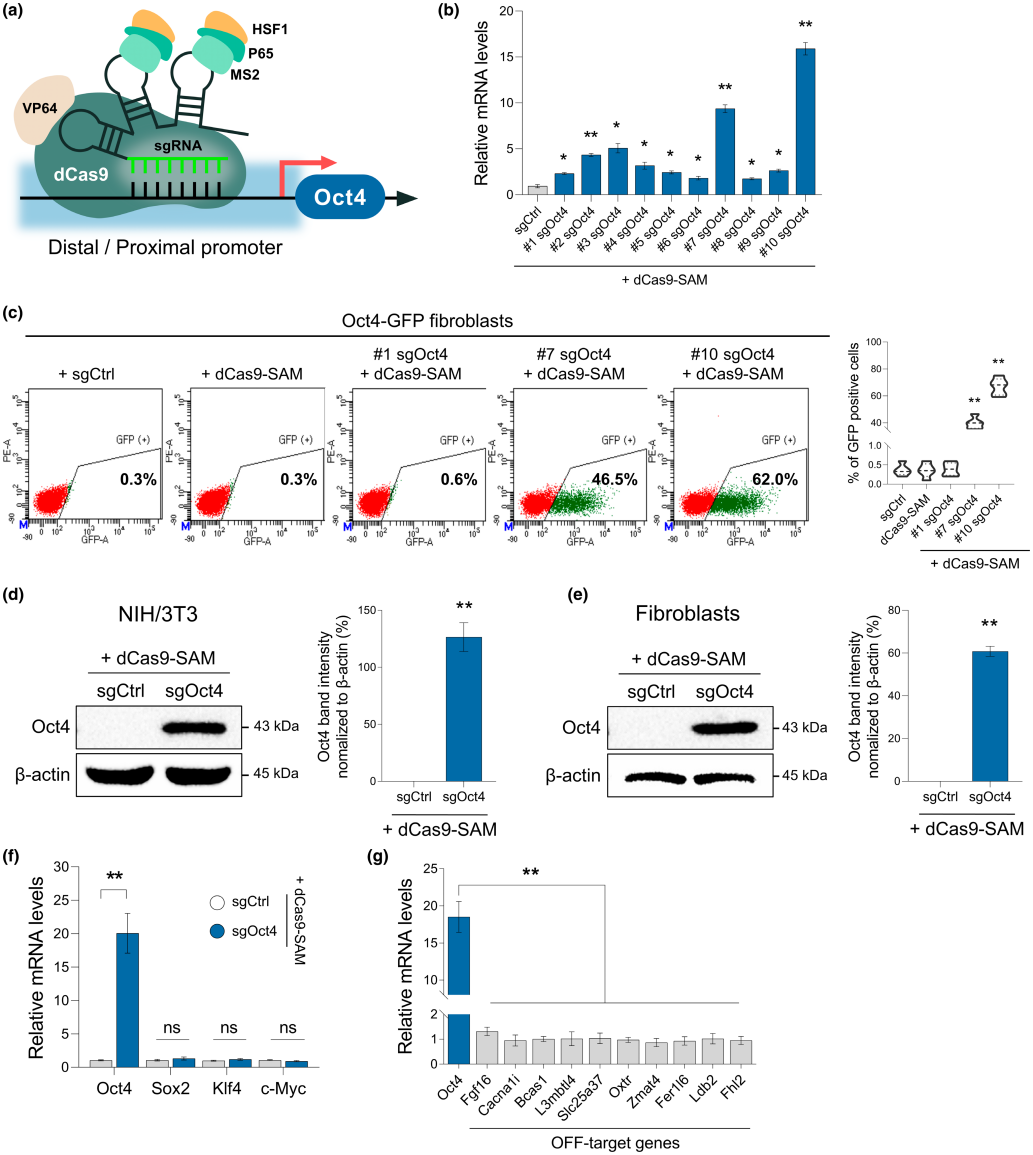
Screening of sgRNAs used for dCas9 activator inducing endogenous Oct4 expression. (a) Scheme depicting the stimulatory effect of Cas9‐VP64‐MS2‐p65‐HSF1 (dCas9‐SAM) with sgRNAs on the transcription of the Oct4 gene with distal and proximal promoter regions. (b) Assessment of the endogenous Oct4 mRNA levels via RT‐qPCR with RNA extracted from NIH/3T3 cells 2 days after treatment with dCas9‐SAM and sgRNAs (Oct4 single‐guide RNA (#1–10 sgOct4) targeting the distal or proximal promoter region of the Oct4 gene and control single‐guide RNA (sgCtrl) targeting the bacterial sequence). Data represent mean ± SEM; one‐way ANOVA, *p < 0.05 and **p < 0.005 (*n* = 5, independent samples per group). (c) Representative flow cytometry plots showing the GFP‐positive cells from Oct4‐GFP mouse tail‐tip fibroblasts (TTFs) treated with either the sgCtrl or #1, #7, #10 sgOct4. Data represent mean ± SEM; one‐way ANOVA, *p < 0.05 and **p < 0.005 (*n* = 5, independent samples per group). (d, e) Western blot analysis of Oct4 and β‐actin in NIH/3T3 cells (d) and Tail‐tip fibroblasts (e) treated with sgCtrl or sgOct4 (Left). The bar chart shows the quantification data (Right). Data represent mean ± SEM; two‐tailed Student's *t* test, *p < 0.05 and **p < 0.005 (*n* = 3, independent samples per group). (f) Assessment of the endogenous Oct4, Sox2, Klf4, and c‐Myc mRNA levels via RT‐qPCR with RNA extracted from TTFs 2 days after treatment with dCas9‐SAM together with the sgCtrl or #10 sgOct4. Data represent mean ± SEM; one‐way ANOVA, *p < 0.05 and **p < 0.005 (*n* = 5, independent samples per group). (g) RT‐qPCR analysis of potential off‐target genes of dCas9‐Oct4 activator in TTFs. Data represent mean ± SEM; one‐way ANOVA, *p < 0.05 and **p < 0.005 (*n* = 5, independent samples per group).

Previous studies have reported that Oct4 mainly activates genes associated with pluripotency and self‐renewal but also simultaneously represses differentiation‐associated genes (Pan et al., 2002). Several lines of evidence have suggested that activation of Oct4 expression by using small‐molecule compounds (Hou et al., 2013) or additional factors is sufficient to initiate cellular reprogramming of somatic cells, suggesting that induction of Oct4 expression alone is sufficient to initiate reprogramming but not sufficient for complete reprogramming to the pluripotent state (Kim et al., 2021). Thus, we evaluated whether activation of the expression of the endogenous Oct4 gene via CRISPR/dCas9‐activator could be used to induce partial reprogramming for cellular rejuvenation. For the optimal induction of endogenous Oct4 expression by the CRISPR/dCas9‐activator, we designed 10 candidate sgRNAs targeting Oct4 distal or proximal promoter regions (Figure 1a; Table S1). Two days after transfection of NIH/3T3 cells with each sgRNA, we confirmed the CRISPR/dCas9‐activator‐mediated induction of endogenous Oct4 expression in the mCherry positive cells, containing the dCas9 activator vector (Figure S1a) and #7 or #10 sgRNA targeting to the Oct4 proximal promoter region identified as the most efficient targeting site for Oct4 transactivation activities (Figure 1b). Moreover, since the endogenous Oct4 gene was directly activated by the CRISPR/dCas9 activator, the dCas9‐mediated Oct4 activation could be quantified via the Oct4‐GFP knock‐in reporter system. We observed that compared with other sgRNAs, #10 sgRNA (targeting 59 bp upstream of the start codon) rendered the highest GFP expression in Oct4‐GFP knock‐in fibroblasts (Figure 1c). Consistently, we further confirmed that Oct4 expression was significantly induced by the dCas9‐Oct4 activator in mouse NIH/3T3 cells and tail‐tip fibroblasts (Figure 1d,e). Also, dCas9‐activator transiently activated Oct4 gene expression (Figure S1b). These results indicated that the dCas9‐Oct4 activator could efficiently activate Oct4 gene expression in mouse cells. Next, we investigated whether other pluripotency genes could be activated by dCas9‐mediated Oct4 activator. Consistent with our previous results, RT‐qPCR analysis showed that only Oct4 expression was induced by the dCas9‐Oct4 activator, and no alteration was observed in Sox2, Klf4, and c‐Myc mRNA levels (Figure 1f). Moreover, to investigate the off‐target effects of the dCas9‐Oct4 activator, we examined 10 off‐target sites that might be targeted by the Oct4 sgRNA sequence and confirmed that neither of these sites exhibited any transcriptional activation (Figure 1g; Table S2). Thus, these results suggested that the dCas9‐Oct4 activator specifically activated the endogenous Oct4 gene expression in mouse somatic cells without off‐target effects.

### 3.2 Efficient epigenetic remodeling of somatic fibroblasts by the dCas9‐Oct4 activator

**FIGURE 2:**
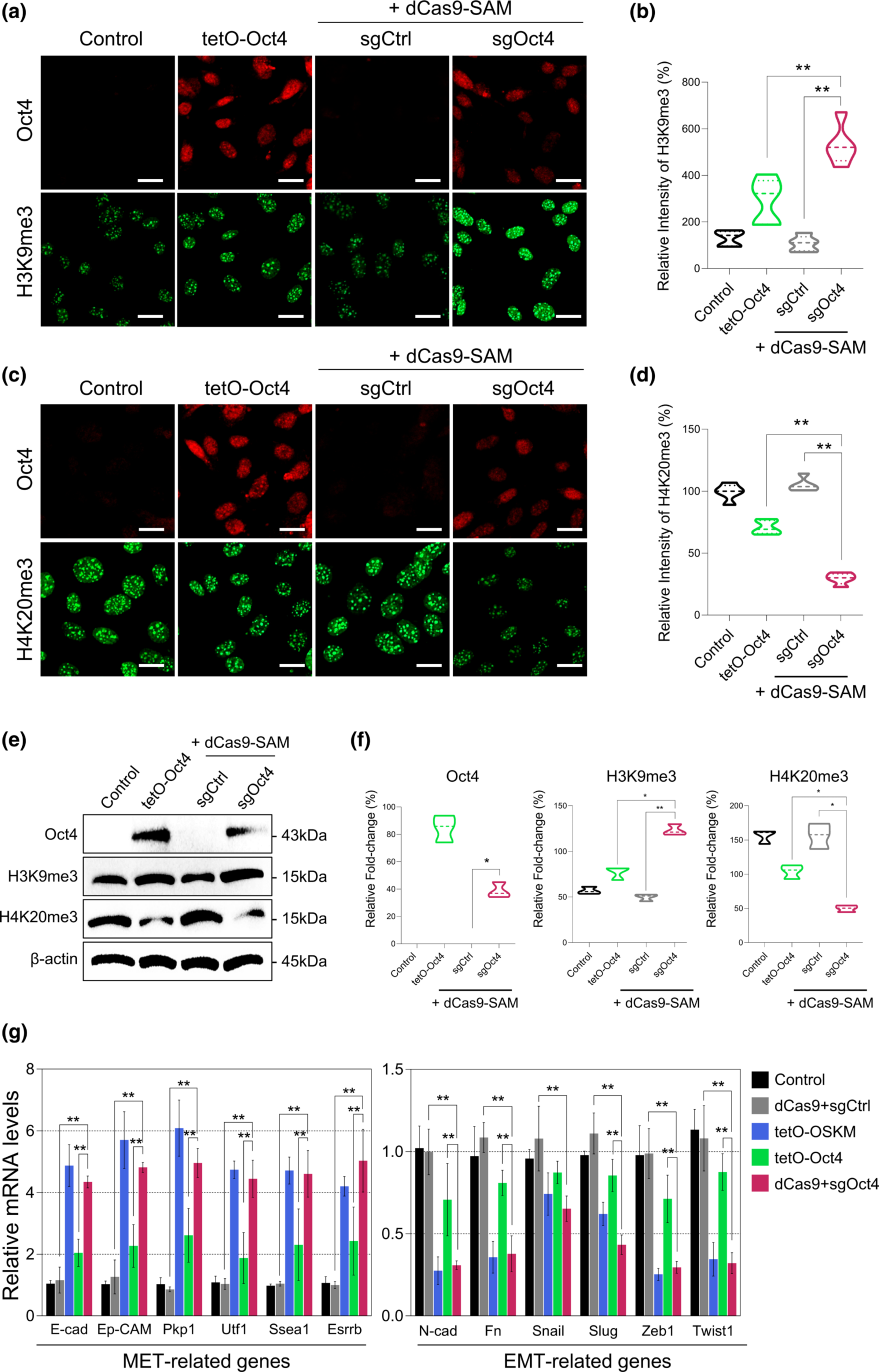
dCas9‐Oct4 activator‐mediated endogenous Oct4 efficiently induced epigenetic remodeling in mouse fibroblasts. (a, c) Immunocytochemistry with Oct4 (red), (a) H3K9me3 (green), and (c) H4K20me3 (green) in mouse Tail‐tip fibroblasts (TTFs) transfected with tetO‐Oct4 or sgOct4. Data represent mean ± SEM; one‐way ANOVA, *p < 0.05 and **p < 0.005 (*n* = 5, independent samples per group). (*n* = 5, Scale bar: 30 μm). (b, d) Quantification of the relative intensity of (b) H3K9me3 and (d) H4K20me3 compared to control. Data represent mean ± SEM; one‐way ANOVA, *p < 0.05 and **p < 0.005 (*n* = 5, independent samples per group). (*n* = 5, Scale bar: 30 μm). (e) Western blot analysis of Oct4, H3K9me3, H4K20me3, and β‐actin in TTFs transfected with tetO‐Oct4 or sgOct4. (f) Quantification of (e) Oct4, H4K20me3, and H3K9me3 levels normalized to the β‐actin levels. Data represent mean ± SEM; one‐way ANOVA, *p < 0.05 and **p < 0.005 (*n* = 3, independent samples per group). (g) RT‐qPCR analysis for genes related to mesenchymal‐to‐epithelial transition (MET), including multiple cell–cell adhesion proteins (E‐cadherin, Ep‐CAM, and the desmosomal protein Pkp1), pluripotency‐associated genes (Utf1 and Esrrb), and embryonic stem cell marker (SSEA1). The mRNAs of various epithelial‐to‐mesenchymal transition‐activating transcription factors (EMT‐ATFs), including mesenchymal markers (Fn and N‐cadherin) and transcriptional repressors (Snail, Slug, Zeb1, and Twist1). Data represent mean ± SEM; two‐way ANOVA, *p < 0.05 and **p < 0.005 (*n* = 3, independent samples per group).

Previous studies have shown that OSKM‐mediated reprogramming involves dramatic epigenetic modifications at the initial stage (Hansson et al., 2012; Polo et al., 2012). Thus, we examined whether this epigenetic remodeling can be efficiently achieved through the CRISPR/dCas9‐mediated transcriptional activation of the endogenous Oct4 gene. To evaluate the effect of the dCas9‐Oct4 activator on the epigenetic modifications, we first assessed the levels of H3K9me3 and H4K20me3, which are involved in maintaining the heterochromatin structure during cellular senescence, in skin fibroblasts. Interestingly, we observed that H3K9me3 was significantly upregulated by the dCas9‐Oct4 activator in the fibroblasts, but only marginally upregulated by exogenous expression of Oct4 (Figure 2a,b,e,f). Consistently, H4K20me3 was significantly downregulated by the dCas9‐Oct4 activator but not by exogenous Oct4 expression (Figure 2c–f).

Next, RT‐qPCR analysis showed that the dCas9‐Oct4 activator increased the mRNA levels of genes associated with the mesenchymal‐to‐epithelial transition (MET), including *E*‐*cadherin*, *Ep*‐*CAM*, *Pkp1*, and the pluripotency genes *Utf1*, *Esrrb*, and *SSEA1* but decreased the mRNA levels of genes associated with the epithelial‐to‐mesenchymal transition (EMT), namely *Fn*, *N*‐*cadherin*, and the transcriptional repressors *Snail*, *Slug*, *Zeb1*, and *Twist1*. Similar to the dCas9‐Oct4 activator, exogenous expression of OSKM caused dramatic alterations in the expression levels of MET/EMT‐related genes, whereas exogenous expression of Oct4 alone was insufficient (Figure 2g). Thus, these results indicated that the dCas9‐Oct4 activator could efficiently induce de‐differentiation of the skin fibroblasts by promoting MET and suppressing EMT via activating endogenous Oct4 expression.

### 3.3 Amelioration of age‐associated hallmarks in aged fibroblasts via dCas9 activator‐mediated transcriptional activation of the endogenous Oct4 gene

**FIGURE 3:**
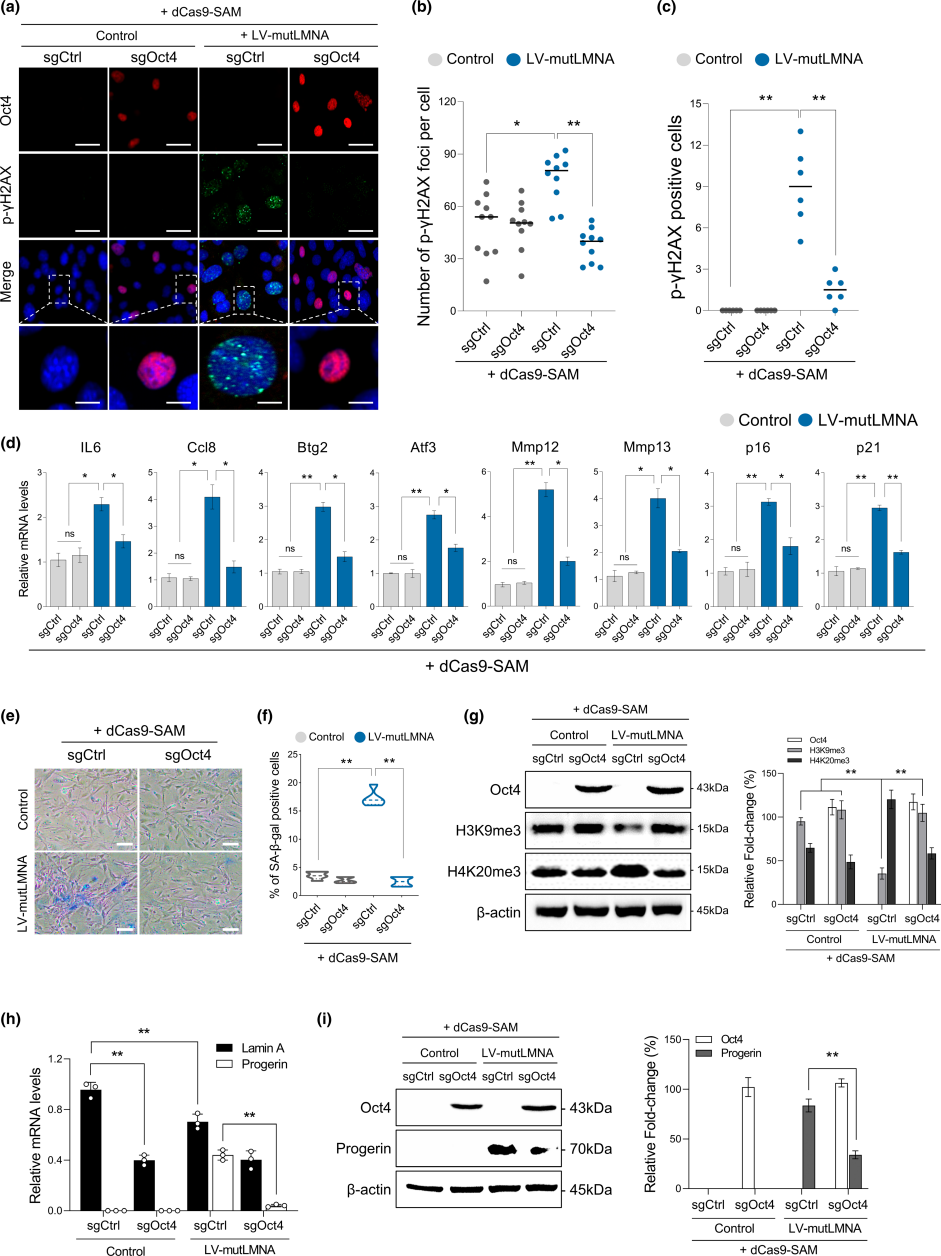
Amelioration of in vitro aged phenotypes by dCas9‐Oct activator‐mediated endogenous Oct4 expression. (a) Immunostaining for Oct4 (red), gamma‐H2AX (green), and DAPI (blue) in dCas9‐Oct4 activator treated TTFs, infected doxycycline (dox)‐inducible lentivirus expressing the mutant LaminA/C (mutLMNA) (Control; non‐treated doxycycline, LV‐mutLMNA; with doxycycline). (Scale bar: 30 μm, 10 μm). (b, c) Quantification of gamma‐H2AX positive cells and nuclear abnormalities. Data represent mean ± SEM; one‐way ANOVA, *p < 0.05 and **p < 0.005 (*n* = 10, *n* = 6, respectively, independent samples per group). (d) RT‐qPCR analysis of the senescence‐associated genes. Total RNA extracted after 2 days of dCas9‐Oct4 activator treatment in TTF with LV‐mutLMNA infection. Data represent mean ± SEM; one‐way ANOVA, *p < 0.05 and **p < 0.005 (*n* = 5, independent samples per group). (e, f) Senescence‐associated‐β‐galactosidase (SA‐β‐gal) staining and quantification of SA‐β‐gal positive cells in after 3 days of dCas9‐Oct4 activator–treated TTFs after LV‐mutLMNA infection. Data represent mean ± SEM; one‐way ANOVA, *p < 0.05 and **p < 0.005 (*n* = 5, independent samples per group). (g) Western blot analysis of H3K9me3 and H4K20me3 after 3 days of dCas9‐Oct4 activator treated TTFs after LV‐mutLMNA infection. The bar chart shows the quantification data (Right). Data represent mean ± SEM; two‐tailed Student's *t* test, *p < 0.05 and **p < 0.005 (*n* = 3, independent samples per group). (h) Assessment of the Lamin A, Progerin mRNA levels via RT‐qPCR from after 3 days of dCas9‐Oct4 activator–treated TTFs derived from LMNA^G608G/G608G^ mice. Data represent mean ± SEM; two‐way ANOVA, *p < 0.05 and **p < 0.005 (*n* = 3, independent samples per group). (i) Western blot analysis of Oct4, Progerin, and β‐actin in tail‐tip fibroblasts LMNA^G608G/G608G^ mice. The bar chart shows the quantification data (Right). Data represent mean ± SEM; two‐tailed Student's *t* test, *p < 0.05 and **p < 0.005 (*n* = 3, independent samples per group).

To determine whether the dCas9‐mediated Oct4 activator could ameliorate hallmarks of aging in vitro, endogenous Oct4 was activated using the dCas9 activator in mouse fibroblasts transduced with a mutant laminA/C (LMNA) (Figure S2a). Given that DNA double‐strand breaks are associated with cellular aging, we first assessed the level of phosphorylated histone gamma‐H2AX, a marker of such breaks, after the activation of the endogenous Oct4 gene. Remarkably, phosphorylated histone gamma‐H2AX was significantly reduced by the dCas9‐Oct4 activator (Figure 3a). Moreover, the number of cells with phosphorylated histone gamma‐H2AX or abnormal nuclei was also significantly reduced as a consequence of dCas9‐mediated activation of endogenous Oct4 expression. (Figure 3b,c). To evaluate the expression patterns of aging‐related genes after dCas9‐mediated Oct4 activation, we conducted RT‐qPCR analysis of the stress response genes *p16* and *p21*, associated with the tumor suppressor pathways, in addition to the senescence‐associated inflammatory signaling genes *Ccl8* and *IL*‐*6*, and the senescence‐related genes *Btg2*, and *Atf3*. Consistent with the previous results (Ocampo et al., 2016), the expression patterns of these aging‐related genes were effectively restored by dCas9‐mediated activation of endogenous Oct4 expression in aged fibroblasts expressing the mutant LMNA (Figure 3d). These results showed that the dCas9‐mediated activation of the endogenous Oct4 gene can efficiently ameliorate aging‐related molecular phenotypes in senescent fibroblasts in vitro. Additionally, to further confirm the anti‐aging effects of the dCas9‐mediated Oct4 activator in mutant LMNA transduced aged fibroblasts, we assessed the age‐associated phenotypes via immunofluorescence assays. Two days after the activation of endogenous Oct4 expression by the dCas9 activator, immunocytochemical analysis revealed that the number of apoptotic cells, evidenced by the expression of cleaved caspase 3, was significantly reduced compared with the control level (Figure S2b,c). Additionally, senescence‐associated beta‐galactosidase positive cells were reduced in aged fibroblasts cells by dCas9‐mediated endogenous Oct4 activation (Figure 3e,f). To assess how long dCas9 activator induce anti‐aging phenotypes is maintained, the number of beta‐galactosidase positive cells in fibroblasts transduced with a mutant LMNA was observed during the time course. We found that the number of beta‐galactosidase positive cells decreased from 3 to 7 days but increased again after 10 days (Figure S2d,e). These results indicated that dCas9‐Oct4 activator‐mediated anti‐aging effects were not permanent if Oct4 expression was discontinued. Moreover, consistent with previous results (Ocampo et al., 2016), we observed decreased H3K9me3 and increased H4K20me3 in fibroblasts upon transduction of mutant LMNA. Further, we demonstrated that Oct4 activation efficiently restored the level of these marks to control in the LMNA mutant infected fibroblasts (Figure 3g). Finally, to examine the possibility of whether dCas9 mediated Oct4‐induction is sufficient for the repression of progerin expression, we accessed the expression of Lamin A and Progerin in Lmna‐mutant fibroblasts after dCas9 mediated Oct4‐induction. We found that effective suppression of Lmna‐mutant progerin by the dCas9‐Oct4 activator (Figure 3h,i) indicating rescued progeria phenotypes by repressing the mutant LMNA gene through endogenous Oct4 induction. These results suggest that CRISPR/dCas9‐mediated transient activation of endogenous Oct4 expression alone can efficiently alleviate the accumulation of age‐associated phenotypes.

### 3.4 CRISPR/dCas9 activator‐mediated endogenous Oct4 activation rescued the HGPS‐associated vascular pathological features and lifespan shortening in progeria mice

**FIGURE 4:**
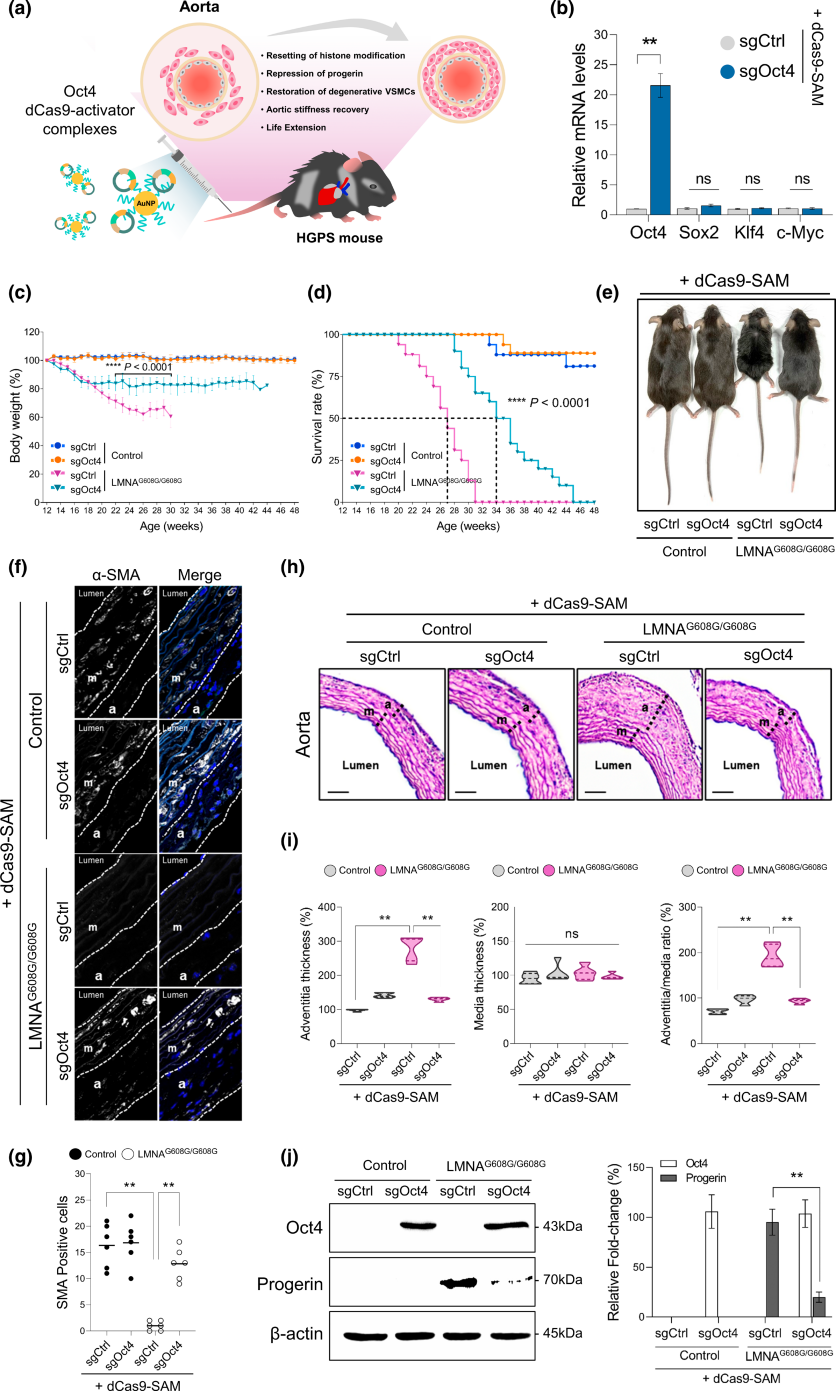
Amelioration of aortic histopathology and lifespan in the progeria mice by dCas9‐Oct4 activator‐mediated endogenous Oct4 expression. (a) Schematic drawing depicting the experimental setting to deliver the AuNPs/dCas9‐Oct4 activator in LMNA^G608G/G608G^. AuNPs/dCas9‐Oct4 activator was delivered via tail‐vein injection (t.v.i.) to a 3‐month‐old LMNA^G608G/G608G^. (b) Oct4, Sox2, Klf4, and c‐Myc mRNA levels were assessed via RT‐qPCR with total RNA extracted at 7 days after t.v.i. (Data represent mean ± SEM; two‐way ANOVA, *p < 0.05 and **p < 0.005 (*n* = 5, independent samples per group). (c) Survival rates of sgCtrl or sgOct4 treated control and LMNA^G608G/G608G^, 7 weeks after AuNPs/dCas9‐Oct4 activator delivery. sgCtrl‐treated mice (Control *n* = 16; LMNA^G608G/G608G^ *n* = 16) and sgOct4 treated mice (Control *n* = 18; LMNA^G608G/G608G^ *n* = 20). ****p < 0.0001 according to log‐rank (Mantel‐Cox) test. (d) Body weight progression of sgCtrl or sgOct4 treated control and LMNA^G608G/G608G^, 7 weeks after AuNPs/dCas9‐Oct4 activator delivery. sgCtrl‐treated mice (Control *n* = 16; LMNA^G608G/G608G^ *n* = 16) and sgOct4 treated mice (Control *n* = 18; LMNA^G608G/G608G^ *n* = 20). ****p < 0.0001 according to two‐way ANOVA with Tukey's post hoc test. (e) Representative image of 5‐month‐old Control (C57BL/6J mouse that lacks the transgene) and 5‐month‐old LMNA^G608G/G608G^ treated sgCtrl and sgOct4, respectively. (f, g) Immunofluorescence staining and quantification of the aorta stained with an anti‐alpha‐smooth muscle actin (α‐SMA) antibody (white) and DAPI (blue). The aorta was collected from sgCtrl or sgOct4 treated control and LMNA^G608G/G608G^, 7 weeks after AuNPs/dCas9‐Oct4 activator delivery (Scale bar: 50 μm). Data represent mean ± SEM; one‐way ANOVA, *p < 0.05 and **p < 0.005 (*n* = 6, independent samples per group). (h) Representative aorta cross‐sections. Images were stained with hematoxylin and eosin. (*n* = 5, Scale bar: 50 μm). (i) Quantification of the adventitia area thickness, median thickness, and ratio of adventitia area/media area. Data represent mean ± SEM; one‐way ANOVA, *p < 0.05 and **p < 0.005 (*n* = 5, independent samples per group). (j) Western blot analysis of Oct4, Progerin, and β‐actin in VSMCs of LMNA^G608G/G608G^. The bar chart shows the quantification data (Right). Data represent mean ± SEM; two‐tailed Student's *t* test, *p < 0.05 and **p < 0.005 (*n* = 3, independent samples per group).

To examine whether dCas9‐mediated endogenous Oct4 activation could ameliorate aging‐related phenotypes in vivo, we initially prepared dCas9‐Oct4 activator nanocomplex with cationic polyethyleneimine (PEI)‐conjugated gold nanoparticles (AuNPs) for transient delivery of sgRNA along with Cas9 protein in vivo. Previously, we have shown this AuNPs/PEI mediated nanocomplexes can be used as efficient and non‐toxic gene carriers for in vivo applications (Chang et al., 2019). The premature progeria mice were injected with AuNPs/dCas9 complexes through the lateral tail vein to activate the endogenous Oct4 gene (Figure 4a). One week after post‐injection, we examined the level of endogenous Oct4 transcription in the aorta of the mice. Consistent with the previous results, we observed significant transcriptional activation of the endogenous Oct4 gene via the dCas9‐activator, whereas other pluripotent factors, namely *Sox2*, *Klf4*, and *c*‐*Myc*, were not affected (Figure 4b). Importantly, to demonstrate the temporal expression of Oct4 in vivo using dCas9 activator system, we examined Oct4 expression in the aorta after systemic delivery of the dCas9 activator. Consistent with previous results, we observed that Oct4 expression in the aorta was significantly elevated at Day 3 and peaked at Day 7 and gradually declined until Day 14 (Figure S3a). Also, we confirmed the expression of Oct4 in other tissues such as kidney, liver, and skin. We observed the Oct4 expression was maintained for about 2 weeks after in vivo delivery of dCas9‐Oct4 activator in these tissues (Figure S3b). Additionally, in order to validate what percentage of cells expressed Oct4 in in vivo tissue, we counted the number of Oct4‐positive cells in the aorta area of the progeria mouse after delivery of dCas9‐Oct4 activator. We observed more than 50% of total cells expressed Oct4 in the aorta area at 5 days after Cas9‐Oct4 activator delivery demonstrating the robust expression of the Oct4 in the adult tissue of progeria mice (Figure S3c).

Moreover, we observed that significant improvements in external appearance after AuNPs/dCas9 mediated Oct4 activation, including a reduction in the spine curvature in the progeria mice treated with the dCas9‐Oct4 activator (Figure 4e). We also monitored the body weight progression and life span of mice treated with the dCas9‐Oct4 activator in comparison with the sgCtrl‐treated mice. We observed that dCas9‐Oct4 activator treatment in progeria mice attenuated the weight loss (Figure 4c) and induced a dramatic increase in the median and maximal lifespans of the animals (Figure 4d). Conversely, the sgCtrl‐treated mice did not show any improvement in the lifespan shortening or weight loss.

Previously, transient expression of OSKM in vivo has been shown to cause tumorigenesis in various tissues (Ohnishi et al., 2014). Thus, we assessed for tumorigenesis upon activation of endogenous Oct4 expression via tail‐vein injection of the dCas9‐activator. We monitored the body weight progression and life span of mice treated with the dCas9‐Oct4 activator in comparison with the exogenous OSKM induction. (Figure S4a,b). Also, we observed that exogenous OSKM induction caused tumorigenesis in multiple organs, whereas no such tumor was observed in the dCas9‐mediated Oct4 induction group (Figure S4c,d). Additionally, RT‐qPCR analysis revealed that tumor‐related genes were not induced in the liver of mice transduced with the dCas9‐Oct4 activator (Figure S4e). A previous study showed the activation of exogenous Oct4 resulted in dysplastic growths in epithelial tissues that are dependent on continuous Oct4 expression (Hochedlinger et al., 2005). Since the dCas9 activator system induced temporally endogenous Oct4 expression in vivo, we did not find any particular dysplasia phenotypes in our mice (Figure S4f). These results indicate that dCas9 activator‐mediated in vivo partial reprogramming does not lead to any negative consequences.

Mice with HGPS mostly die due to cardiovascular ailments similar to those seen in old age. Progerin‐induced vascular smooth muscle cell (VSMC) death is a key driver of mortality in old age (Hamczyk et al., 2018). Additionally, this mouse model exhibits a loss of VSMCs in aortic vessel walls, which together contribute to aortic stiffening and impairment of cardiac function (Murtada et al., 2020). To assess the physiological consequences of dCas9‐Oct4 activator treatment, we histologically analyzed the aorta of the HGPS mouse model. Importantly, sgOct4 with dCas9 activator treated progeria mice rescued the degeneration of vascular smooth muscle cells (VSMCs) compared to sgCtrl‐treated mice (Figure 4f,g). Notably, the progeria mice treated with the sgOct4‐guided dCas9‐activator were completely rescued of the adventitial and median thickness phenotypes compared with those of the sgCtrl‐treated progeria mice (Figure 4h,i). To examine the possibility of whether dCas9‐Oct4 activator is sufficient for the repression of progerin expression in progeria mice, we accessed the expression in the aorta of the progeria mouse after dCas9 mediated Oct4‐induction. We found that effective suppression of Lmna‐mutant progerin by the dCas9‐Oct4 activator rescued progeria phenotypes by repressing the mutant LMNA gene through Oct4 induction (Figure 4j).

We further conducted RT‐qPCR analysis of the aging‐related genes in progeria mice after transduction of the Oct4 dCas9‐activator. The expression levels of these age‐associated genes were effectively restored by activation of the endogenous Oct4 gene in mice aorta (Figure S5a). These results indicate that in vivo transient expression of single endogenous Oct4 expression significantly improved the aging‐related degeneration phenotype of VSMCs and aging‐related vascular stiffening. To further validate the effect of dCas9‐activator system in various organs, we first assessed the expression of senescence‐associated genes such as Btg2, p21, IL6, Mmp12, and Atl3 in kidney, spleen, muscle, lung, and skin. Consistent with previous results, Btg2, p21, IL6, Mmp12, and Atl3 were significantly downregulated by the dCas9‐Oct4 activator in these tissues of progeria mice (Figure S5b). Also, we observed the rejuvenated phenotypes in the epidermis thickness in the skin and diameter of renal tubules of kidney by the dCas9 activator‐mediated Oct4 activation (Figure S5c,d). In addition, to investigate effects of dCas9‐Oct4 activator in wild‐type aging, we examined the expression of senescence‐associated genes of the skin, aorta, spleen, kidney, and liver in the Oct4 activated aged wild‐type mice (Figure S6a). We observed that significant downregulation of senescence‐associated genes in the aged tissues of the 24‐month‐old mice by the dCas9‐Oct4 activator indicating that dCas9 activator‐mediated temporal expression of Oct4 restores age‐related gene expression in aged wild‐type mice. Notably, we have previously shown that endogenous Oct4 expression by the Oct4 dCas9‐activator strongly leads to epigenetic alteration of H3K9me3 and H4K20me3. Subsequently, we conducted immunoblotting on dCas9‐Oct4 activator treated aorta from progeria mice. Immunoblotting analysis showed a significant increase in H3K9me3 and decrease in H4K20me3 in the sgOct4‐treated HGPS mice (Figure S5e). This result indicated that epigenetic remodeling via CRISPR/dCas9‐induced activation of endogenous Oct4 expression improves aging‐related cardiovascular abnormalities.

## 4 DISCUSSION

In recent years, numerous studies have demonstrated that partial reprogramming by transient expression of Oct4, Sox2, Klf4, and c‐Myc (termed OSKM) rejuvenates cells in vitro and ameliorates aging‐related phenotypes in vivo in the tissues of the treated animals (Ocampo et al., 2016). These results have shown that partial cellular reprogramming can reduce the deleterious effects of aging by delaying or reversing aging‐related phenotypes and can also increase regenerative capacity (Taguchi & Yamada, 2017). Thus, cellular rejuvenation reprogramming approaches promise new strategies as cell replacement therapy for aging‐related degenerative diseases. In this study, we report that transient CRISPR/dCas9‐mediated transcriptional activation of a single gene, Oct4, could induce partial reprogramming of differentiated cells to a rejuvenated state in vitro and in vivo in a mouse model of premature aging. Oct4 is known to be central to the molecular machinery governing pluripotency and plays a critical role in re‐establishing pluripotency in somatic cells for the proper reprogramming to pluripotency (Shi & Jin, 2010), suggesting that endogenous expression of Oct4 can be an effective strategy for cellular rejuvenation reprogramming (Kim et al., 2021). Consistent with this notion, we observed that CRISPR/dCas9‐induced expression of the endogenous Oct4 gene efficiently ameliorates aging‐related epigenetic patterns, such as the perturbed levels of H3K9me3 and H4K20me3. Previous studies suggested that the decreased H3K9me3 and increased H4K20me3 were closely associated with cellular aged phenotypes (Ocampo et al., 2016). Thus, these perturbations can be explained by two reasons: First, dCas9 activator‐mediated endogenous Oct4 induction might trigger more efficiently rejuvenation reprogramming since endogenous Oct4 expression during pluripotent reprogramming was known to be critical for inducing pluripotency and re‐establishing the epigenetic landscape (Kim et al., 2021; Pardo et al., 2010). Moreover, the heterochromatin reorganization depends on establishment of the endogenous pluripotency gene expression and their transcriptional networks (Fussner et al., 2011). Thus, dCas9 activator‐mediated endogenous Oct4 expression might affect the age‐related heterochromatin reorganization which can directly increase H3K9me3 and decrease H4K20me3. Moreover, we found that CRISPR/dCas9 mediated endogenous Oct4 induction effectively suppressed the expression of mutant progerin of the aorta. It has been shown previously that progerin expression was suppressed during the OSKM induced pluripotent reprogramming (Liu et al., 2011). Given that Oct4 induction inhibits progerin expression, our data suggested that endogenous Oct4 induction affects the anti‐aging phenotypes most likely through reduced progerin accumulation by restoring the aberrant nuclear envelope architecture. Thus, while Oct4 induction may have additional effects in the reprogramming progress for rejuvenation, our study indicates that only transient reduced progerin expression may be useful for extend longevity and provided more efficient therapeutic strategies in progeric syndromes based on LMNA mutation.

In addition, our results showed that transcriptionally transient activation of the endogenous Oct4 gene increased their survival and decreased aging‐related vascular deterioration in the progeria mouse. The rejuvenation effect depends on the continuous expression of Oct4, and from this point of view, the continuous expression of endogenous Oct4 through the dCas9‐Oct4 activator can be a very effective method of inhibiting progerin expression. Taken together, our results suggested that the CRISPR/dCas9 activator can be used to effectively induce endogenous Oct4 expression and applied as a potential treatment of aging‐related diseases in the future.

Importantly, to demonstrate the temporal expression of Oct4 in vivo using dCas9 activator system, we examined Oct4 expression in the aorta after systemic delivery of the dCas9 activator. Consistent with previous results, we observed that Oct4 expression in the aorta was significantly elevated at Day 3 and peaked at Day 7 and gradually declined until Day 14 (Figure S3a). Also, we confirmed the expression of Oct4 in other tissues such as kidney, liver, and skin. We observed the Oct4 expression was maintained for about 2 weeks after in vivo delivery of dCas9‐Oct4 activator in these tissues (Figure S3b). Additionally, in order to validate what percentage of cells expressed Oct4 in in vivo tissue, we counted the number of Oct4‐positive cells in the aorta area of the progeria mouse after delivery of dCas9‐Oct4 activator. We observed more than 50% of total cells expressed Oct4 in the aorta area at 5 days after Cas9‐Oct4 activator delivery demonstrating the robust expression of the Oct4 in the adult tissue of progeria mice (Figure S3c).

There are several limitations to the classic gene‐overexpression systems to induce transgenes for partial reprogramming. For example, the size of transgene vectors, in particular, is limited, but this limitation can be overcome by using a CRISPR/dCas9 activator system, where gene targets are determined by the corresponding sgRNA. Moreover, it is difficult to rapidly control endogenous gene expression. Unlike exogenous overexpression, CRISPR/dCas9‐mediated activation of endogenous genes is rapid, the expression level remains high even for a long time in vitro and in vivo, and the effect can be reversible. Since the ability of reversible induction of gene expression is vital for partial reprogramming, CRISPR/dCas9 activator–mediated manipulation of gene expression is an attractive strategy, especially for the treatment of aging‐related degenerative diseases.

Importantly, we observed that rejuvenating somatic cells by transiently activating endogenous Oct4 expression had a lower tumorigenesis risk than OSKM expression. For instance, several lines of evidence in transgenic mice carrying inducible OSKM have suggested that ubiquitous overexpression of OSKM has deleterious effects on organs. Although the cyclic or transient expression of OSKM can induce partial reprogramming and improve aging‐related phenotypes, the use of the oncogenic factors Klf4 and c‐Myc during the reprogramming process has the risk of inducing oncogenesis. Moreover, Oct4 induction by dCas9 activator might be relatively lower than that induced by TetO‐OSKM based lentivirus in vivo. Thus, due to this relatively low expression of Oct4 by Cas9 activator, the dCas9‐Oct4‐activating system did not induce tumors, while TetO‐OSKM induced several tumors. In addition, although reprogramming through transient expression of a single exogenous factor, such as Oct4 without Klf4 and c‐Myc, has been reported, the transient overexpression of Oct4 was relatively inefficient in activating the gene network for partial reprogramming. Therefore, partial reprogramming technologies that do not use oncogenic factors need to be developed for safe rejuvenation strategies.

We found that the CRISPR/dCas9 activator could efficiently activate the endogenous Oct4 and pluripotency genes for successful partial reprogramming. To our knowledge, this is the first report of in vivo rejuvenation reprogramming via CRISPR/dCas9‐activator induction of endogenous Oct4 expression. The success of CRISPR/dCas9‐mediated efficient partial reprogramming opens an avenue to generate safe and efficient methods for rejuvenation and eliminates the potential risks of the use of oncogenic factors.

## AUTHOR CONTRIBUTIONS

Junyeop Kim: Conceptualization, Methodology, Writing—Original draft, Review & Editing, Yerim Hwang: Validation, Visualization, Methodology, Investigation, Writing—Original draft, Review & Editing, Sumin Kim: Investigation, Methodology, Yujung Chang: Investigation, Methodology, Yunkyung Kim: Investigation, Methodology Youngeun Kwon: Investigation, Methodology, Jongpil Kim: Supervision, Project administration, Funding acquisition, Writing—original draft, Review & Editing.

## CONFLICT OF INTEREST STATEMENT

The authors declare no competing financial interests.

## Supporting information


Appendix S1

Data S1–S20

## Data Availability

All data needed to evaluate the conclusions in the paper are present in the paper and/or the Supporting Information. Additional data related to this paper may be requested from the authors.
