# Supplementary material for: Transcriptional activation of endogenous Oct4 via the CRISPR/dCas9 activator ameliorates Hutchinson‐Gilford progeria syndrome in mice
Source: Aging Cell. 2023 Mar 25;22(6):e13825. doi: 10.1111/acel.13825 (PMC10265166; doi:10.1111/acel.13825)
Supplement: Supplementary file 1 — Appendix S1 [file ACEL-22-e13825-s002.docx]

Supplementary information to

**Transcriptional activation of endogenous Oct4 via the CRISPR/dCas9 activator ameliorates Hutchinson-Gilford Progeria Syndrome in mice**

Junyeop Kim, Yerim Hwang, Sumin Kim, Yujung Chang, Yunkyung Kim, Youngeun Kwon, Jongpil Kim





**Table S1. sgRNA sequences for targeting endogenous Oct4 promoter locus**

**(a)** Binding positions of the ten sgRNAs used for targeting dCas9-SAM to the Oct4 promoter locus.





**Table S2.** **List of potential top10 off-target sites homologous to Oct4 sgRNA #10.**

**(a)** List of potential off-target sites homologous to #10 sgOct4. PAM sequence (NGG) is underlined in red. (ON; on-target, OFF; off-target)





**Figure S1. Validation of Oct4 mRNA expression by dCas9-Oct4 activator**

**(a)** Co-expression of Oct4 and mCherry-tag in NIH/3T3 demonstrates transduction of Oct4-dCas9 activator. **(b)** RT-qPCR analysis of Oct4 at different time points after dCas9-Oct4 activator transfection. Data represent mean ± SEM; one-way ANOVA, *p < 0.05 and **p < 0.005 (n = 5, independent samples per group).

**Figure S2. Amelioration of *in vitro* aged phenotypes by dCas9-Oct activator**

**(a)** Schematic representation of the induction of Oct4 in TTFs via dCas9-Oct4 activator system. TTFs were infected with LV-mutLMNA prior to dCas9-Oct4 activator transfection and treated with doxycycline for 2 days. **(b and c)** Immunostaining and quantification of cleaved-caspase3 (green) positive cells in AuNPs/dCas9-Oct4 activator–treated TTFs after LV-mutLMNA infection at 2 days post-transfection, compared with that of the control. (n = 6, Scale bar: 30µm). **(d and e)** Senescence-associated-β-galactosidase (SA-β-gal) staining and quantification of SA-β-gal positive cells at different time points after dCas9-Oct4 activator treated TTFs after LV-mutLMNA infection. Data represent mean ± SEM; one-way ANOVA, *p < 0.05 and **p < 0.005 (n = 5, independent samples per group).





**Figure S3. Validation of in vivo Oct4 expression by AuNPs/dCas9 activator system.**

**(a)** Assessment of the endogenous Oct4 mRNA level via RT-qPCR from AuNPs/dCas9-Oct4 activator treated LMMA^G608G/G608G^ mice aorta. Data represent mean ± SEM; one-way ANOVA, *p < 0.05 and **p < 0.005 (n = 5, independent samples per group). **(b)** RT-qPCR analysis of the endogenous Oct4 mRNA level in liver, spleen, kidney, lung, skin and muscle of dCas9-Oct4 activator treated LMMA^G608G/G608G^ mice. Total RNA extracted from 2 weeks after first transfection of AuNPs/dCas9-Oct4 activator. Data represent mean ± SEM; two-way ANOVA, *p < 0.05 and **p < 0.005 (n = 3, independent samples per group). **(c)** Immunostaining for Oct4 and DAPI in aorta, liver, spleen, kidney, lung, skin and muscle of AuNPs/dCas9-Oct4 activator treated LMMA^G608G/G608G^ mice (Data not shown). Quantification of Oct4 positive cells per DAPI (n=6, n=5 respectively, independent samples per group). Data represent mean ± SEM; two-way ANOVA, *p < 0.05 and **p < 0.005 (n = 3, independent samples per group).

**Figure S4. Assessment of the tumorigenesis upon activation of endogenous Oct4 expression by dCas9-Oct4 activator**

**(a)** Body weight progression of Control, tetO-OSKM and sgOct4 treated wild type mice, 7 weeks after AuNPs/dCas9-Oct4 activator delivery. Control (n=6), sgOct4 treated mice (n=9) and tetO-OSKM treated mice (n=8). ****p <0.0001 according to two-way ANOVA with Tukey’s post hoc test. **(b)** Survival rate of Control, tetO-OSKM and sgOct4 treated control and LMNA^G608G/G608G^. Control (n=6), sgOct4 treated mice (n=9) and tetO-OSKM treated mice (n=8). ****p <0.0001 according to log-rank (Mantel-Cox) test. **(c and d)** Photo show that no primary tumor formation (arrow) observed in dCas9-Oct4 activator treated mice. Data represent mean ± SEM; two-way ANOVA, *p < 0.05 and **p < 0.005 (n = 3, independent samples per group). **(e)** RT-qPCR analysis of tumor-related genes in tetO-OSKM and sgOct4 treated mice via tail vein injection. Data represent mean ± SEM; one-way ANOVA, *p < 0.05 and **p < 0.005 (n = 5, independent samples per group). **(f)** Representative cross sections of liver, kidney and skin from 5-month old LMNA^G608G/G608G^ treated with sgCtrl or sgOct4. Images were stained with hematoxylin and eosin. (n =5, scale bar = 50μm).





**Figure S5. Improvement of *in vivo* aged phenotypes in progeria mice tissues.**

**(a)** RT-qPCR analysis of senescence-associated genes in the Aorta from dCas9-Oct4 activator treated LMMA^G608G/G608G^ mice. Data represent mean ± SEM; one-way ANOVA, *p < 0.05 and **p < 0.005 (n = 5, independent samples per group). **(b)** RT-qPCR analysis of the senescence-associated genes in dCas9-Oct4 activator treated LMMA^G608G/G608G^ mice tissues. Data represent mean ± SEM; two-way ANOVA, *p < 0.05 and **p < 0.005 (n = 3, independent samples per group). **(c)** Representative cross sections of kidney and skin from sgCtrl or sgOct4 treated control and LMNA^G608G/G608G^. Images were stained with hematoxylin and eosin. (n =5, scale bar = 50μm). Arrows depict decreased diameter of renal tubules and epidermis thickness in the progeria mice. **(d)** Quantification of the diameter of renal tubles and thickness of epidermis. Data represent mean Data represent mean ± SEM; one-way ANOVA, *p < 0.05 and **p < 0.005 (n = 5, independent samples per group). **(e)** Western blot analysis of H3K9me3 and H4K20me3 in the aorta. The aorta was collected from sgCtrl or sgOct4 treated control and LMNA^G608G/G608G^. The bar chart shows the quantification data (Right). Data represent mean ± SEM; two-tailed Student’s t-test, *p < 0.05 and **p < 0.005 (n = 3, independent samples per group).

**

**

**Figure S6. Amelioration of aged phenotypes in wild-type mice by dCas9-Oct4 activator**

**(a)** RT-qPCR analysis of the senescence-associated genes in dCas9-Oct4 activator treated tissues of 24-month old wild type mice. Data represent mean ± SEM; two-way ANOVA, *p < 0.05 and **p < 0.005 (n = 3, independent samples per group).
