# Supplementary material for: Transcriptional activation of endogenous Oct4 via the CRISPR/dCas9 activator ameliorates Hutchinson‐Gilford progeria syndrome in mice
Source: Aging Cell. 2023 Mar 25;22(6):e13825. doi: 10.1111/acel.13825 (PMC10265166; doi:10.1111/acel.13825)
Supplement: Supplementary file 2 — Data S1–S20 [file ACEL-22-e13825-s001.pdf]

Supporting data 1. List of qRT-PCR primers for Pluripotency associated genes.

| Pluripotency associated genes | | |
|---|---|---|
| Oct4 | F | GGCTTCAGACTTCGCCTTCT |
| | R | TGGAAGCTTAGCCAGGTTCG |
| Sox2 | F | TTTGTCCGAGACCGAGAAGC |
| | R | CTCCGGGAAGCGTGTACTTA |
| Klf4 | F | GCACACCTGCGAACTCACAC |
| | R | CCGTCCCAGTCACAGTGGTAA |
| c-Myc | F | ACCACCAGCAGCGACTCTGA |
| | R | TGCCTCTTCTCCACAGACACC |

Supporting data 2. List of qRT-PCR primers for MET/EMT associated genes.

| MET/EMT associated genes | | |
|---|---|---|
| E-cadherin | F | GCCCTGCCAATCCCGATGAAA |
| | R | GGGGTCAGTATCAGCCGCT |
| EP-CAM | F | GAAGGGGCGATCCAGAACAA |
| | R | TGGTCGTAGGGGCTTTCTCT |
| Pkp1 | F | CACGAGCAGAAGCAGCTACT |
| | R | TGCGGCTTGCCTTGATTTTC |
| Utf1 | F | ACCAGATCCGCCAACTCATG |
| | R | GCAGACTTCGTCGTGGAAGA |
| Ssea1 | F | CGTGTCTGTGGACGTGTTTG |
| | R | GCACGAAGCGCTCATAGTTG |
| Esrrb | F | TTTCTGGAACCCATGGAGAG |
| | R | AGCCAGCACCTCCTTCTACA |
| N-cad | F | AGCCCGGTTTCACTTGAGAG |
| | R | CATCCGCATCAATGGCAGTG |
| FN | F | GACACGTGGAGCAAGAAGGA |
| | R | CCACTGCATTCCCACAGAGT |
| Slug | F | CATCCTTGGGGCGTGTAAGT |
| | R | AGTGCAGCTGCTTGTGTTTG |
| Snail1 | F | CCACTGCAACCGTGCTTTT |
| | R | CACATCCGAGTGGGTTTGG |
| Zeb1 | F | ACAAGACACCGCCGTCATTT |
| | R | GCAGGTGAGCAACTGGGAAA |
| Twist1 | F | CGGGTCATGGCTAACGTG |
| | R | CAGCTTGCCATCTTGGAGTC |

Supporting data 3. List of qRT-PCR primers for Senescence associated genes.

| Senescence associated genes | | |
|---|---|---|
| p16 | F | CGTGAACATGTTGTTGAGGC |
| | R | GCAGAAGAGCTGCTACGTGA |
| p21 | F | CGGTGTCAGAGTCTAGGGGA |
| | R | ATCACCAGGATTGGACATGG |
| Atf3 | F | CTCTGGCCGTTCTCTGGA |
| | R | GGTCGCACTGACTTCTGAGG |
| Btg2 | F | GCGAGCAGAGACTCAAGGTT |
| | R | TAGCCAGAACCTTTGGATGG |
| Mmp13 | F | TGATGAAACCTGGACAAGCA |
| | R | GGTCCTTGGAGTGATCCAGA |
| Mmp12 | F | CGCCTCTCTGCTGATGACAT |
| | R | GGGATGCTTGGCCATATGGA |
| IL6 | F | TGATGCACTTGCAGAAAACA |
| | R | ACCAGAGGAAATTTTCAATAGGC |
| Ccl8 | F | TTTGCCTGCTGCTCATAGCT |
| | R | TCACTGACCCACTTCTGTGT |

Supporting data 4. List of qRT-PCR primers for Tumor associated genes.

| Tumor associated genes | | |
| --- | --- | --- |
| afp | F | GCTGCTCAGTACGACAAGGT |
| | R | GGGCCAGCTTCTGAATCTCA |
| egfr | F | TGTGGGCCTGACTACTACGA |
| | R | GAGTGCGCGTGAAAGAATCC |
| gpc3 | F | GGCAAGGAACGGGATGAAGA |
| | R | CCGTCACCAGAGCTTCCAAT |
| cd19 | F | GAGAGGCACGTGAAGGTCAT |
| | R | GGGAGGCGTCACTTTGAAGA |
| cd24a | F | GCTTCTGGCACTGCTCCTA |
| | R | GAGATGTAGAAGAGAGAGAGAGAGA |
| cd44 | F | AGCCCCTCCTGAAGAAGACT |
| | R | GCTTTCTGGGGTGCTCTTCT |

Supporting data 5. List of antibodies used in this study.

| Antibodies | Source | Cat. No | Application | Dilution |
|---|---|---|---|---|
| **H3K9me3** | abcam | ab8898 | WB | 500:1 |
| **H4K20me3** | abcam | ab78517 | WB | 500:1 |
| **Oct4** | abcam | ab18976 | WB | 1000:1 |
| **Oct4** | santacruz | sc-5279 | WB | 500:1 |
| **beta actin** | abfrontier | LF-PA0207 | WB | 1000:1 |
| **H3** | abcam | ab6002 | WB | 500:1 |
| **Oct4** | abcam | ab18976 | ICC | 1000:1 |
| **Oct4** | santacruz | sc-5279 | ICC | 500:1 |
| **phospho-gammaH2AX** | Cell signaling | 9718 | ICC | 500:1 |
| **cleaved caspase-3** | Cell signaling | 9661 | ICC | 500:1 |
| **α-SMA** | Invitrogen | 14-9760-82 | IHC | 500:1 |

Supporting data 6. Information of body weight for Figure 4c

| weeks | 12 | 13 | 14 | 15 | 16 | 17 | 18 | 19 | 20 | 21 | 22 | 23 | 24 | 25 | 26 | 27 | 28 | 29 | 30 | 31 | 32 | 33 | 34 | 35 | 36 | 37 | 38 | 39 | 40 | 41 | 42 | 43 | 44 | 45 | 46 | 47 | 48 |
|---|---|---|---|---|---|---|---|---|---|---|---|---|---|---|---|---|---|---|---|---|---|---|---|---|---|---|---|---|---|---|---|---|---|---|---|---|---|
| C57BL/6J +sgCtrl | 100 | 103 | 102 | 102 | 101 | 102 | 101 | 101 | 101 | 101 | 101 | 101 | 101 | 101 | 99 | 100 | 98 | 98 | 98 | 102 | 100 | 101 | 102 | 101 | 102 | 103 | 102 | 101 | 100 | 99 | 98 | 99 | 102 | 103 | 103 | 102 | 104 |
| | 100 | 103 | 102 | 102 | 101 | 102 | 103 | 102 | 102 | 102 | 101 | 102 | 103 | 102 | 102 | 100 | 100 | 100 | 100 | 102 | 102 | 102 | 103 | 103 | 102 | 103 | 103 | 103 | 102 | 103 | 103 | 102 | 101 | 101 | 100 | 100 | 101 |
| | 100 | 103 | 101 | 101 | 100 | 102 | 103 | 102 | 102 | 102 | 102 | 103 | 103 | 104 | 103 | 102 | 101 | 100 | 99 | 101 | 100 | 100 | 101 | 101 | 101 | 102 | 102 | 102 | 101 | 102 | 102 | 101 | 101 | 101 | 101 | 100 | 101 |
| | 100 | 102 | 102 | 103 | 102 | 104 | 105 | 102 | 103 | 103 | 102 | 102 | 103 | 103 | 104 | 102 | 102 | 102 | 102 | 102 | 102 | 102 | 101 | 100 | 100 | 99 | 100 | 100 | 99 | 99 | 99 | 99 | 99 | 100 | 100 | 100 | 100 |
| | 100 | 101 | 101 | 102 | 101 | 103 | 104 | 103 | 103 | 104 | 104 | 105 | 105 | 106 | 105 | 103 | 103 | 103 | 102 | 104 | 104 | 104 | 105 | 105 | 104 | 105 | 105 | 104 | 104 | 104 | 104 | 103 | 102 | 102 | 101 | 100 | 101 |
| | 100 | 102 | 102 | 100 | 100 | 102 | 103 | 102 | 103 | 104 | 105 | 106 | 107 | 106 | 105 | 103 | 102 | 101 | 100 | 100 | 99 | 100 | 101 | 100 | 100 | 101 | 102 | 102 | 101 | 102 | 101 | 100 | 100 | 100 | 100 | 100 | 100 |
| | 100 | 102 | 102 | 103 | 101 | 102 | 102 | 102 | 104 | 104 | 104 | 103 | 102 | 103 | 102 | 102 | 102 | 101 | 101 | 103 | 103 | 103 | 104 | 103 | 103 | 102 | 103 | 102 | 102 | 102 | 101 | 101 | 101 | 102 | 102 | 101 | 102 |
| | 100 | 103 | 102 | 104 | 103 | 104 | 104 | 103 | 102 | 103 | 102 | 103 | 103 | 104 | 104 | 102 | 102 | 102 | 102 | 103 | 103 | 103 | 102 | 101 | 100 | 101 | 102 | 101 | 101 | 102 | 101 | 101 | 100 | 100 | 100 | 100 | 100 |
| | 100 | 103 | 103 | 102 | 102 | 103 | 104 | 103 | 102 | 103 | 103 | 102 | 103 | 103 | 104 | 103 | 102 | 101 | 100 | 102 | 102 | 103 | 104 | 104 | 103 | 102 | 102 | 101 | 101 | 101 | 101 | 100 | 99 | 100 | 99 | 99 | 99 |
| | 100 | 105 | 104 | 104 | 103 | 104 | 105 | 104 | 104 | 104 | 103 | 104 | 105 | 104 | 104 | 102 | 102 | 102 | 102 | 104 | 104 | 104 | 105 | 105 | 104 | 105 | 105 | 105 | 104 | 105 | 105 | 104 | 103 | 103 | 102 | 102 | 103 |
| | 100 | 103 | 103 | 104 | 103 | 105 | 106 | 103 | 104 | 104 | 103 | 103 | 104 | 104 | 105 | 103 | 103 | 103 | 103 | 103 | 103 | 103 | 102 | 101 | 101 | 100 | 101 | 101 | 100 | 100 | 100 | 100 | 100 | 101 | 101 | 101 | 101 |
| | 100 | 104 | 104 | 102 | 102 | 104 | 105 | 104 | 105 | 106 | 107 | 108 | 109 | 108 | 107 | 105 | 104 | 103 | 102 | 102 | 101 | 102 | 103 | 102 | 102 | 103 | 104 | 104 | 103 | 104 | 103 | 102 | 102 | 102 | 102 | 102 | 102 |
| | 100 | 102 | 101 | 103 | 102 | 103 | 103 | 102 | 101 | 102 | 101 | 102 | 102 | 103 | 103 | 101 | 101 | 101 | 101 | 102 | 102 | 102 | 101 | 100 | 99 | 100 | 101 | 100 | 100 | 101 | 100 | 100 | 99 | 99 | 99 | 99 | 99 |
| | 100 | 102 | 101 | 101 | 100 | 101 | 100 | 100 | 100 | 100 | 100 | 100 | 100 | 100 | 98 | 99 | 97 | 97 | 97 | 101 | 99 | 100 | 101 | 100 | 101 | 102 | 101 | 100 | 99 | 98 | 97 | 98 | | | | | |
| | 100 | 104 | 104 | 102 | 102 | 104 | 104 | 103 | 102 | 103 | 102 | 103 | 102 | 103 | 103 | 101 | 101 | 101 | 100 | 100 | 100 | 100 | | | | | | | | | | | | | | | |
| | 100 | 103 | 103 | 101 | 101 | 103 | 103 | 102 | 101 | 102 | 101 | 102 | 101 | 102 | 102 | 100 | 100 | 100 | 99 | 99 | 99 | | | | | | | | | | | | | | | | |
| C57BL/6J +Oct4 | 100 | 102 | 102 | 101 | 101 | 102 | 101 | 100 | 101 | 101 | 99 | 101 | 101 | 99 | 97 | 100 | 97 | 98 | 97 | 102 | 99 | 99 | 102 | 101 | 100 | 101 | 100 | 100 | 103 | 98 | 96 | 102 | 100 | 101 | 106 | 100 | 107 |
| | 100 | 102 | 101 | 102 | 100 | 101 | 106 | 101 | 100 | 105 | 99 | 101 | 101 | 101 | 102 | 98 | 99 | 99 | 103 | 100 | 100 | 101 | 101 | 101 | 102 | 101 | 102 | 101 | 105 | 102 | 102 | 102 | 99 | 100 | 100 | 99 | 100 |
| | 100 | 106 | 101 | 101 | 100 | 102 | 103 | 101 | 102 | 105 | 102 | 101 | 103 | 104 | 101 | 105 | 101 | 100 | 97 | 100 | 100 | 99 | 101 | 100 | 101 | 101 | 101 | 105 | 101 | 105 | 101 | 104 | 100 | 99 | 100 | 99 | 101 |
| | 100 | 102 | 101 | 102 | 102 | 103 | 105 | 100 | 102 | 101 | 100 | 101 | 103 | 103 | 103 | 102 | 101 | 101 | 105 | 100 | 102 | 101 | 99 | 99 | 98 | 98 | 103 | 100 | 98 | 97 | 98 | 99 | 102 | 100 | 100 | 103 | 99 |
| | 100 | 104 | 101 | 102 | 101 | 103 | 103 | 103 | 102 | 103 | 103 | 108 | 108 | 105 | 105 | 103 | 101 | 102 | 102 | 103 | 103 | 103 | 103 | 105 | 107 | 104 | 105 | 103 | 103 | 103 | 103 | 103 | 100 | 101 | 104 | 99 | 100 |
| | 100 | 102 | 101 | 100 | 99 | 101 | 103 | 101 | 101 | 103 | 103 | 106 | 105 | 105 | 105 | 102 | 102 | 101 | 103 | 98 | 98 | 103 | 104 | 100 | 100 | 101 | 102 | 101 | 100 | 100 | 99 | 99 | 103 | 99 | 103 | 103 | 99 |
| | 100 | 102 | 101 | 103 | 100 | 102 | 105 | 102 | 103 | 102 | 103 | 103 | 105 | 102 | 105 | 102 | 102 | 101 | 99 | 103 | 102 | 101 | 102 | 102 | 102 | 105 | 103 | 102 | 102 | 102 | 104 | 101 | 101 | 100 | 101 | 100 | 102 |
| | 100 | 106 | 102 | 104 | 106 | 104 | 104 | 103 | 102 | 102 | 101 | 103 | 103 | 104 | 104 | 102 | 101 | 105 | 100 | 102 | 101 | 106 | 102 | 100 | 99 | 101 | 105 | 99 | 100 | 102 | 99 | 99 | 100 | 103 | 98 | 98 | 98 |
| | 100 | 103 | 102 | 102 | 102 | 106 | 107 | 101 | 100 | 101 | 103 | 101 | 102 | 101 | 107 | 102 | 100 | 99 | 103 | 100 | 101 | 101 | 104 | 103 | 103 | 102 | 102 | 100 | 99 | 101 | 100 | 98 | 98 | 98 | 99 | 102 | 98 |
| | 100 | 101 | 100 | 101 | 99 | 100 | 105 | 100 | 99 | 104 | 98 | 100 | 100 | 100 | 101 | 97 | 98 | 98 | 102 | 99 | 99 | 100 | 100 | 100 | 101 | 100 | 101 | 100 | 104 | 101 | 101 | 101 | 98 | 99 | 99 | 98 | 99 |
| | 100 | 104 | 99 | 99 | 98 | 100 | 101 | 99 | 100 | 103 | 100 | 99 | 101 | 102 | 99 | 103 | 99 | 98 | 95 | 98 | 98 | 97 | 99 | 98 | 99 | 99 | 99 | 103 | 99 | 103 | 99 | 102 | 98 | 97 | 98 | 97 | 99 |
| | 100 | 104 | 103 | 104 | 104 | 105 | 107 | 102 | 104 | 103 | 102 | 103 | 105 | 105 | 105 | 104 | 103 | 103 | 107 | 102 | 104 | 103 | 101 | 101 | 100 | 100 | 105 | 102 | 100 | 99 | 100 | 101 | 104 | 102 | 102 | 105 | 101 |
| | 100 | 105 | 102 | 103 | 102 | 104 | 104 | 104 | 103 | 104 | 104 | 109 | 109 | 106 | 106 | 104 | 102 | 103 | 103 | 104 | 104 | 104 | 104 | 106 | 108 | 105 | 106 | 104 | 104 | 104 | 104 | 104 | 101 | 102 | 105 | 100 | 101 |
| | 100 | 101 | 100 | 99 | 98 | 100 | 102 | 100 | 100 | 102 | 102 | 105 | 104 | 104 | 104 | 101 | 101 | 100 | 102 | 97 | 97 | 102 | 103 | 99 | 99 | 100 | 101 | 100 | 99 | 99 | 98 | 98 | 102 | 98 | 102 | 102 | 98 |
| | 100 | 100 | 99 | 101 | 98 | 100 | 103 | 100 | 101 | 100 | 101 | 101 | 103 | 100 | 103 | 100 | 100 | 99 | 97 | 101 | 100 | 99 | 100 | 100 | 100 | 103 | 101 | 100 | 100 | 100 | 102 | 99 | 99 | 98 | 99 | 98 | 100 |
| | 100 | 105 | 101 | 103 | 105 | 103 | 103 | 102 | 101 | 101 | 100 | 102 | 102 | 103 | 103 | 101 | 100 | 104 | 99 | 101 | 100 | 105 | 101 | 99 | 98 | 100 | 104 | 98 | 99 | 101 | 98 | 98 | 99 | 102 | 97 | 97 | 97 |
| | 100 | 103 | 103 | 102 | 102 | 103 | 102 | 101 | 102 | 102 | 100 | 102 | 102 | 100 | 98 | 101 | 98 | 99 | 98 | 103 | 100 | 100 | 103 | 102 | | | | | | | | | | | | | |
| | 100 | 103 | 102 | 100 | 101 | 103 | 101 | 102 | 99 | 102 | 99 | 102 | 99 | 102 | 102 | 98 | 98 | 100 | 97 | 99 | 99 | 97 | 98 | | | | | | | | | | | | | | |
| LMNA G608G/G608G +sgCtrl | 100 | 103 | 100 | 99 | 94 | 89 | 86 | 81 | 76 | 73 | 72 | 70 | 67 | 67 | 68 | 70 | 71 | 71 | 66 | | | | | | | | | | | | | | | | | | |
| | 100 | 101 | 99 | 96 | 91 | 86 | 83 | 80 | 77 | 72 | 67 | 65 | 62 | 62 | 57 | 59 | 60 | 60 | 55 | | | | | | | | | | | | | | | | | | |
| | 100 | 101 | 98 | 97 | 95 | 92 | 89 | 84 | 79 | 76 | 71 | 69 | 64 | 64 | 61 | 67 | 64 | 64 | | | | | | | | | | | | | | | | | | | |
| | 100 | 104 | 101 | 100 | 95 | 90 | 87 | 82 | 77 | 74 | 73 | 71 | 68 | 68 | 69 | 71 | 72 | 72 | | | | | | | | | | | | | | | | | | | |
| | 100 | 100 | 98 | 95 | 90 | 85 | 82 | 79 | 76 | 71 | 66 | 64 | 61 | 61 | 56 | 58 | 59 | | | | | | | | | | | | | | | | | | | | |
| | 100 | 100 | 98 | 93 | 88 | 85 | 82 | 77 | 72 | 67 | 66 | 61 | 67 | 64 | 61 | 67 | | | | | | | | | | | | | | | | | | | | | |
| | 100 | 99 | 96 | 95 | 93 | 90 | 87 | 82 | 77 | 74 | 69 | 67 | 62 | 62 | 59 | 65 | | | | | | | | | | | | | | | | | | | | | |
| | 100 | 100 | 97 | 94 | 92 | 87 | 84 | 79 | 76 | 74 | 69 | 67 | 64 | 59 | 65 | | | | | | | | | | | | | | | | | | | | | | |
| | 100 | 101 | 99 | 94 | 89 | 86 | 83 | 78 | 73 | 68 | 67 | 62 | 68 | 65 | 62 | | | | | | | | | | | | | | | | | | | | | | |
| | 100 | 99 | 97 | 96 | 91 | 88 | 85 | 82 | 77 | 75 | 78 | 76 | 70 | 70 | | | | | | | | | | | | | | | | | | | | | | | |
| | 100 | 98 | 96 | 95 | 90 | 87 | 84 | 81 | 76 | 74 | 77 | 75 | 69 | 69 | | | | | | | | | | | | | | | | | | | | | | | |
| | 100 | 98 | 95 | 92 | 90 | 85 | 82 | 77 | 74 | 72 | 67 | 65 | 62 | | | | | | | | | | | | | | | | | | | | | | | | |
| | 100 | 98 | 95 | 94 | 91 | 89 | 92 | 91 | 86 | 81 | 80 | 78 | | | | | | | | | | | | | | | | | | | | | | | | | |
| | 100 | 98 | 96 | 95 | 93 | 90 | 87 | 82 | 77 | 75 | 74 | | | | | | | | | | | | | | | | | | | | | | | | | | |
| | 100 | 98 | 95 | 94 | 91 | 86 | 83 | 80 | 80 | | | | | | | | | | | | | | | | | | | | | | | | | | | | |
| | 100 | 99 | 97 | 96 | 91 | 86 | 81 | 80 | | | | | | | | | | | | | | | | | | | | | | | | | | | | | |
| LMNA G608G/G608G +sgOct4 | 100 | 99 | 95 | 94 | 93 | 89 | 86 | 85 | 79 | 82 | 81 | 86 | 85 | 86 | 88 | 87 | 87 | 74 | 70 | 68 | 74 | 76 | 78 | 78 | 80 | 80 | 79 | 81 | 80 | 80 | 81 | 79 | 82 | | | | |
| | 100 | 100 | 96 | 95 | 94 | 90 | 87 | 86 | 80 | 83 | 82 | 87 | 86 | 87 | 89 | 88 | 88 | 75 | 71 | 69 | 75 | 77 | 79 | 79 | 81 | 81 | 80 | 82 | 81 | 81 | 82 | 80 | 83 | | | | |
| | 100 | 97 | 94 | 95 | 91 | 87 | 86 | 88 | 89 | 92 | 95 | 96 | 92 | 91 | 90 | 88 | 89 | 89 | 90 | 90 | 89 | 88 | 85 | 86 | 87 | 88 | 86 | 87 | 88 | 90 | 90 | | | | | | |
| | 100 | 98 | 95 | 96 | 92 | 88 | 87 | 89 | 90 | 93 | 96 | 97 | 93 | 92 | 91 | 89 | 90 | 90 | 91 | 91 | 90 | 89 | 86 | 87 | 88 | 89 | 87 | 88 | 89 | 91 | | | | | | | |
| | 100 | 96 | 92 | 93 | 86 | 80 | 79 | 78 | 76 | 79 | 77 | 80 | 79 | 78 | 81 | 83 | 85 | 86 | 89 | 88 | 87 | 87 | 86 | 86 | 86 | 85 | 87 | 87 | | | | | | | | | |
| | 100 | 94 | 90 | 91 | 84 | 78 | 77 | 76 | 74 | 77 | 75 | 78 | 77 | 76 | 79 | 81 | 83 | 84 | 87 | 86 | 85 | 85 | 84 | 84 | 84 | 83 | | | | | | | | | | | |
| | 100 | 95 | 94 | 94 | 85 | 83 | 78 | 79 | 81 | 84 | 84 | 88 | 82 | 82 | 80 | 79 | 79 | 78 | 80 | 80 | 78 | 77 | 76 | 75 | 76 | | | | | | | | | | | | |
| | 100 | 97 | 95 | 94 | 93 | 89 | 89 | 89 | 91 | 91 | 88 | 87 | 83 | 81 | 84 | 85 | 86 | 86 | 86 | 86 | 86 | 86 | 87 | 84 | | | | | | | | | | | | | |
| | 100 | 93 | 92 | 92 | 83 | 81 | 76 | 77 | 79 | 82 | 82 | 86 | 80 | 80 | 78 | 77 | 77 | 76 | 78 | 78 | 78 | 76 | 75 | 74 | 73 | | | | | | | | | | | | | |
| | 100 | 99 | 97 | 96 | 95 | 91 | 91 | 91 | 93 | 93 | 90 | 89 | 85 | 83 | 86 | 87 | 88 | 88 | 88 | 88 | 88 | 88 | 89 | 86 | | | | | | | | | | | | | |
| | 100 | 101 | 95 | 94 | 90 | 85 | 87 | 88 | 88 | 88 | 85 | 85 | 78 | 80 | 86 | 85 | 85 | 85 | 85 | 85 | 85 | 85 | | | | | | | | | | | | | | | |
| | 100 | 100 | 94 | 93 | 89 | 84 | 86 | 87 | 87 | 87 | 84 | 84 | 77 | 79 | 85 | 84 | 84 | 84 | 84 | 84 | 84 | 84 | | | | | | | | | | | | | | | |
| | 100 | 96 | 92 | 93 | 85 | 85 | 85 | 85 | 84 | 84 | 84 | 84 | 80 | 79 | 79 | 79 | 78 | 77 | 80 | 80 | 81 | | | | | | | | | | | | | | | | |
| | 100 | 95 | 91 | 93 | 88 | 80 | 78 | 75 | 79 | 81 | 81 | 86 | 83 | 86 | 86 | 87 | 86 | 85 | 84 | | | | | | | | | | | | | | | | | | |
| | 100 | 102 | 98 | 94 | 95 | 87 | 87 | 87 | 87 | 86 | 86 | 86 | 86 | 82 | 81 | 81 | 81 | 80 | 79 | | | | | | | | | | | | | | | | | | |
| | 100 | 96 | 92 | 94 | 89 | 81 | 79 | 76 | 80 | 82 | 82 | 87 | 84 | 87 | 87 | 88 | 87 | 86 | | | | | | | | | | | | | | | | | | | |
| | 100 | 97 | 91 | 90 | 88 | 82 | 86 | 82 | 85 | 85 | 84 | 87 | 80 | 82 | 82 | 82 | 82 | | | | | | | | | | | | | | | | | | | | |
| | 100 | 96 | 90 | 89 | 87 | 81 | 85 | 81 | 84 | 84 | 83 | 86 | 79 | 81 | 81 | 81 | 81 | | | | | | | | | | | | | | | | | | | | |
| | 100 | 98 | 95 | 95 | 91 | 87 | 87 | 85 | 83 | 80 | 78 | 75 | 70 | 68 | 72 | 71 | | | | | | | | | | | | | | | | | | | | | |
| | 100 | 99 | 96 | 96 | 92 | 88 | 88 | 86 | 84 | 81 | 79 | 76 | 71 | 69 | 73 | 72 | | | | | | | | | | | | | | | | | | | | | |

Supporting data 7. Information of survival rate for Figure 4d.

| weeks | 12 | 13 | 14 | 15 | 16 | 17 | 18 | 19 | 20 | 21 | 22 | 23 | 24 | 25 | 26 | 27 | 28 | 29 | 30 | 31 | 32 | 33 | 34 | 35 | 36 | 37 | 38 | 39 | 40 | 41 | 42 | 43 | 44 | 45 | 46 | 47 | 48 |
|---|---|---|---|---|---|---|---|---|---|---|---|---|---|---|---|---|---|---|---|---|---|---|---|---|---|---|---|---|---|---|---|---|---|---|---|---|---|
| C57BL/6J + sgCtrl | 100 | 100 | 100 | 100 | 100 | 100 | 100 | 100 | 100 | 100 | 100 | 100 | 100 | 100 | 100 | 100 | 100 | 100 | 100 | 100 | 100 | 94 | 88 | 88 | 88 | 88 | 88 | 88 | 88 | 88 | 88 | 88 | 81 | 81 | 81 | 81 | 81 |
| C57BL/6J + Oct4 | 100 | 100 | 100 | 100 | 100 | 100 | 100 | 100 | 100 | 100 | 100 | 100 | 100 | 100 | 100 | 100 | 100 | 100 | 100 | 100 | 100 | 100 | 100 | 94 | 89 | 89 | 89 | 89 | 89 | 89 | 89 | 89 | 89 | 89 | 89 | 89 | 89 |
| LMNA G608G/G608G + sgCtrl | 100 | 100 | 100 | 100 | 100 | 100 | 100 | 100 | 94 | 88 | 88 | 81 | 75 | 69 | 56 | 44 | 31 | 25 | 13 | 0 | 0 | 0 | 0 | 0 | 0 | 0 | 0 | 0 | 0 | 0 | 0 | 0 | 0 | 0 | 0 | 0 | 0 |
| LMNA G608G/G608G + sgOct4 | 100 | 100 | 100 | 100 | 100 | 100 | 100 | 100 | 100 | 100 | 100 | 100 | 100 | 100 | 100 | 100 | 90 | 80 | 75 | 65 | 65 | 60 | 50 | 50 | 35 | 30 | 25 | 25 | 20 | 20 | 15 | 10 | 10 | 0 | 0 | 0 | 0 |

Supporting data 8. Information of body weight for Figure s4a.

| weeks | 20 | 21 | 22 | 23 | 24 | 25 | 26 | 27 | 28 | 29 | 30 | 31 | 32 | 33 | 34 | 35 | 36 |
|---|---|---|---|---|---|---|---|---|---|---|---|---|---|---|---|---|---|
| **C57BL/6J** | 100 | 100 | 100 | 100 | 100 | 100 | 98 | 99 | 97 | 97 | 97 | 101 | 99 | 100 | 101 | 100 | 101 |
| | 100 | 103 | 102 | 103 | 104 | 103 | 103 | 101 | 101 | 101 | 101 | 103 | 103 | 103 | 104 | 104 | 103 |
| | 100 | 100 | 100 | 100 | 101 | 101 | 102 | 101 | 100 | 99 | 98 | 97 | 99 | 98 | 98 | 99 | 99 |
| | 100 | 105 | 106 | 106 | 107 | 107 | 108 | 107 | 105 | 105 | 105 | 104 | 106 | 106 | 106 | 107 | 107 |
| | 100 | 104 | 103 | 104 | 105 | 104 | 104 | 102 | 102 | 102 | 102 | 104 | 104 | 104 | 105 | 105 | 104 |
| | 100 | 99 | 99 | 99 | 100 | 100 | 101 | 100 | 99 | 98 | 97 | 96 | 98 | | | | |
| **C57BL/6J + tetO-OSKM** | 100 | 102 | 102 | 101 | 96 | 91 | 88 | 83 | 78 | 75 | 74 | 72 | | | | | |
| | 100 | 103 | 101 | 98 | 93 | 88 | 85 | 82 | 79 | 74 | | | | | | | |
| | 100 | 99 | 103 | 100 | 99 | 94 | 89 | 86 | | | | | | | | | |
| | 100 | 102 | 103 | 101 | 96 | 91 | 88 | | | | | | | | | | |
| | 100 | 101 | 99 | 97 | 96 | 91 | 88 | | | | | | | | | | |
| | 100 | 101 | 100 | 97 | 96 | 93 | 91 | | | | | | | | | | |
| | 100 | 100 | 98 | 95 | 94 | | | | | | | | | | | | |
| | 100 | 98 | 96 | | | | | | | | | | | | | | |
| **C57BL/6J + sgOct4** | 100 | 102 | 102 | 102 | 102 | 102 | 102 | 100 | 101 | 99 | 99 | 99 | 103 | 101 | 102 | 103 | 102 |
| | 100 | 104 | 104 | 103 | 104 | 105 | 104 | 104 | 102 | 102 | 102 | 102 | 104 | 104 | 104 | 105 | 105 |
| | 100 | 100 | 100 | 100 | 101 | 101 | 102 | 101 | 100 | 99 | 98 | 97 | 99 | 98 | 98 | 99 | 99 |
| | 100 | 102 | 102 | 101 | 101 | 102 | 102 | 103 | 101 | 101 | 101 | 101 | 101 | 101 | 101 | 100 | 99 |
| | 100 | 105 | 106 | 107 | 108 | 109 | 108 | 107 | 105 | 104 | 103 | 102 | 102 | 101 | 102 | 103 | 102 |
| | 100 | 100 | 101 | 101 | 100 | 101 | 101 | 102 | 101 | 100 | 99 | 98 | 100 | 100 | 101 | 102 | 102 |
| | 100 | 105 | 105 | 104 | 104 | 105 | 105 | 106 | 104 | 104 | 104 | 104 | 104 | 104 | 104 | 103 | 102 |
| | 100 | 100 | 101 | 100 | 101 | 101 | 102 | 102 | 100 | 100 | 100 | 100 | 101 | 101 | 101 | 100 | 99 |
| | 100 | 100 | 101 | 100 | 101 | 100 | 101 | 101 | 99 | 99 | 99 | 98 | 98 | 98 | 98 | | |

Supporting data 9. Information of survival rate for Figure s4b.

| weeks | 20 | 21 | 22 | 23 | 24 | 25 | 26 | 27 | 28 | 29 | 30 | 31 | 32 | 33 | 34 | 35 | 36 |
|---|---|---|---|---|---|---|---|---|---|---|---|---|---|---|---|---|---|
| **C57BL/6J** | 100 | 100 | 100 | 100 | 100 | 100 | 100 | 100 | 100 | 100 | 100 | 100 | 100 | 83 | 83 | 83 | 83 |
| **C57BL/6J + tetO-OSKM** | 100 | 100 | 100 | 88 | 88 | 75 | 75 | 38 | 25 | 25 | 13 | 13 | | | | | |
| **C57BL/6J + sgOct4** | 100 | 100 | 100 | 100 | 100 | 100 | 100 | 100 | 100 | 100 | 100 | 100 | 100 | 100 | 100 | 89 | 89 |

Supporting data 10. Nomalized values of qRT-PCR for Figure 1b.

| sgCtrl | #1 sgOct4 | #2 sgOct4 | #3 sgOct4 | #4 sgOct4 | #5 sgOct4 | #6 sgOct4 | #7 sgOct4 | #8 sgOct4 | #9 sgOct4 | #10 sgOct4 |
|---|---|---|---|---|---|---|---|---|---|---|
| 0.742262 | 2.342327 | 4.301856 | 5.50475 | 2.957965 | 2.4477 | 1.647865 | 9.76763 | 1.6320412 | 2.622753 | 15.6538 |
| 1.071773 | 2.275568 | 4.507625 | 5.271153 | 3.604696 | 2.645378 | 2.04786 | 9.409158 | 1.812568 | 2.8101746 | 16.6699 |
| 1 | 2.406775 | 4.27965 | 4.5013364 | 3.017856 | 2.307814 | 1.8077896 | 8.920071 | 1.815675 | 2.530108 | 15.39231 |

Supporting data 11. Percentage of GFP-positive cells for Figure 1c.

**Oct4-GFP fibroblasts**

| **FACS** | sgCtrl | dCas9-SAM | sgOct4 dCas9-SAM | sgOct4 dCas9-SAM | sgOct4 dCas9-SAM |
|---|---|---|---|---|---|
| % of GFP positive cells | 0.3 | 0.3 | 0.6 | 46.5 | 62 |
| | 0.2 | 0.1 | 0.2 | 39.8 | 59.2 |
| | 0.6 | 0.6 | 0.5 | 40.1 | 75.1 |
| | 0.41 | 0.45 | 0.26 | 35.2 | 68.1 |
| | 0.33 | 0.35 | 0.39 | 36.5 | 69.9 |

Supporting data 12. Nomalized values of qRT-PCR for Figure 1g.

| | off target genes | | | | | | | | | |
|---|---|---|---|---|---|---|---|---|---|---|
| **Oct4** | **Fgf16** | **Cacna1i** | **Bcas1** | **L3mbtl4** | **Slc25a37** | **Oxtr** | **Zmat4** | **Fer1l6** | **Ldb2** | **Fhl2** |
| 17.6485 | 1.2123 | 0.8541 | 0.9123 | 1.2165 | 0.765 | 1.1023 | 0.765 | 1.213 | 1.051 | 0.7136 |
| 15.94561 | 1.5315 | 0.6123 | 0.9132 | 0.849 | 0.8651 | 0.951 | 0.6564 | 0.851 | 1.2561 | 0.9321 |
| 18.1965 | 1.451 | 1.056 | 1.0165 | 0.6156 | 1.1532 | 0.8465 | 1.0654 | 0.7646 | 0.7231 | 1.1315 |
| 19.156 | 1.2165 | 1.06531 | 1.056 | 1.165 | 1.156 | 0.9165 | 0.8513 | 0.9123 | 0.91563 | 0.91532 |
| 21.564 | 1.151463 | 1.15614 | 1.1561 | 1.26451 | 1.24561 | 1.0561 | 0.991523 | 0.91546 | 1.154631 | 1.05641 |

Supporting data 13. Nomalized values of media/adventitia thickness for Figure 1i.

| media thickness | | | |
|---|---|---|---|
| sgCtrl | sgOct4 | sgCtrl | sgOct4 |
| 100 | 102.3244 | 118.966 | 96.44944 |
| 105.7857 | 126.131 | 105.62 | 105.7857 |
| 95.52948 | 96.88318 | 103.3068 | 94.42895 |
| 87.35743 | 96.04439 | 91.70091 | 95.61395 |
| 87.77949 | 94.76657 | 96.54771 | 98.69066 |

| adventitia thickness | | | |
|---|---|---|---|
| sgCtrl | sgOct4 | sgCtrl | sgOct4 |
| 100 | 132.8077 | 306.4969 | 132.774 |
| 100.1709 | 137.1177 | 309.0377 | 131.2864 |
| 92.14882 | 138.8356 | 232.2231 | 120.5627 |
| 98.80506 | 149.6214 | 305.9564 | 136.0271 |
| 96.8286 | 148.0319 | 253.2057 | 127.9831 |

Supporting data 14. Relative Oct4 Fold-change (%) for Figure 1d

| +dCas9-SAM | |
|---|---|
| sgCtrl | sgOct4 |
| 4.65E-06 | 58.22019 |
| 2.03E-05 | 62.8363 |
| 1.02E-05 | 61.39476 |

Supporting data 15. Relative Oct4 Fold-change (%) for Figure 1e

| +dCas9-SAM | |
|---|---|
| sgCtrl | sgOct4 |
| 2.11E-05 | 115.113 |
| 1.71E-05 | 124.1799 |
| 7.68E-07 | 140.0695 |

Supporting data 16. Relative Fold-change (%) for Figure 2g

**Relative Oct4 Fold-change (%)**

| | | +dCas9-SAM | |
|---|---|---|---|
| Control | tetO-Oct4 | sgCtrl | sgOct4 |
| 0 | 93.66303 | 0 | 34.16397 |
| 0.000105 | 85.88445 | 0 | 36.90443 |
| 0 | 74.12825 | 0.00025 | 45.12888 |

**Relative H3K9me3 Fold-change (%)**

| | | +dCas9-SAM | |
|---|---|---|---|
| Control | tetO-Oct4 | sgCtrl | sgOct4 |
| 55.93909 | 81.13181 | 52.65607 | 121.1265 |
| 61.36612 | 81.41228 | 51.02837 | 129.8925 |
| 53.46345 | 68.62669 | 45.3175 | 118.4518 |

**Relative H4K20me3 Fold-change (%)**

| | | +dCas9-SAM | |
|---|---|---|---|
| Control | tetO-Oct4 | sgCtrl | sgOct4 |
| 161.0837 | 106.0501 | 173.9499 | 54.35622 |
| 162.36 | 113.1785 | 157.7912 | 44.68404 |
| 144.3245 | 93.09694 | 136.9252 | 50.38627 |

Supporting data 17. Relative Fold-change (%) for Figure 3g

**Relative Oct4 Fold-change (%)**

| +dCas9-SAM | | | |
|---|---|---|---|
| Control | | LV-mutLMNA | |
| sgCtrl | sgOct4 | sgCtrl | sgOct4 |
| 0 | 103.1643 | 0 | 126.9911 |
| 0 | 121.123 | 0 | 109.567 |
| 0 | 110.31 | 0 | 116.452 |

**Relative H3K9me3 Fold-change (%)**

| +dCas9-SAM | | | |
|---|---|---|---|
| Control | | LV-mutLMNA | |
| sgCtrl | sgOct4 | sgCtrl | sgOct4 |
| 99.36072 | 100.1628 | 37.88716 | 114.4885 |
| 91.25 | 105.452 | 40.112 | 105.75 |
| 95.152 | 120.42 | 28.853 | 95.123 |

**Relative H4K20me3 Fold-change (%)**

| +dCas9-SAM | | | |
|---|---|---|---|
| Control | | LV-mutLMNA | |
| sgCtrl | sgOct4 | sgCtrl | sgOct4 |
| 64.2616 | 55.828 | 109.8866 | 52.14469 |
| 70.245 | 50.846 | 120.524 | 58.452 |
| 60.246 | 40.425 | 131.1245 | 65.425 |

Supporting data 18. Relative Fold-change (%) for Figure 3i

**Relative Oct4 Fold-change (%)**

| +dCas9-SAM | | | |
|---|---|---|---|
| Control | | LV-mutLMNA | |
| sgCtrl | sgOct4 | sgCtrl | sgOct4 |
| 0 | 91.60793 | 0 | 107.0718 |
| 0 | 105.425 | 0 | 110.0874 |
| 0 | 110.115 | 0 | 102.2413 |

**Relative Progerin Fold-change (%)**

| +dCas9-SAM | | | |
|---|---|---|---|
| Control | | LV-mutLMNA | |
| sgCtrl | sgOct4 | sgCtrl | sgOct4 |
| 0 | 0 | 83.98898 | 34.0252 |
| 0 | 0 | 90.58432 | 38.45321 |
| 0 | 0 | 77.54214 | 30.4524 |

Supporting data 19. Relative Fold-change (%) for Figure 4j

**Relative Oct4 Fold-change (%)**

| +dCas9-SAM | | | |
|---|---|---|---|
| Control | | $LMNA^{G608G/G608G}$ | |
| sgCtrl | sgOct4 | sgCtrl | sgOct4 |
| 0 | 87.29171 | 0 | 118.866 |
| 0 | 120.5426 | 0 | 100.8887 |
| 0 | 110.7635 | 0 | 92.4563 |

**Relative Progerin Fold-change (%)**

| +dCas9-SAM | | | |
|---|---|---|---|
| Control | | $LMNA^{G608G/G608G}$ | |
| sgCtrl | sgOct4 | sgCtrl | sgOct4 |
| 0 | 0 | 85.42762 | 14.81404 |
| 0 | 0 | 90.7653 | 20.82535 |
| 0 | 0 | 110.5673 | 25.75652 |

Supporting data 20. Relative Fold-change (%) for Figure S5e

**Relative Oct4 Fold-change (%)**

| +dCas9-SAM | | | |
|---|---|---|---|
| Control | | $LMNA^{G608G/G608G}$ | |
| sgCtrl | sgOct4 | sgCtrl | sgOct4 |
| 0 | 116.1559 | 0 | 63.66557 |
| 0 | 68.76534 | 0 | 120.8746 |
| 0 | 115.8765 | 0 | 110.3658 |

**Relative H3K9me3 Fold-change (%)**

| +dCas9-SAM | | | |
|---|---|---|---|
| Control | | $LMNA^{G608G/G608G}$ | |
| sgCtrl | sgOct4 | sgCtrl | sgOct4 |
| 59.92022 | 81.56102 | 21.69532 | 95.82388 |
| 65.36853 | 90.86745 | 17.87366 | 80.2456 |
| 70.87645 | 88.87463 | 29.8746 | 78.17754 |

**Relative H4K20me3 Fold-change (%)**

| +dCas9-SAM | | | |
|---|---|---|---|
| Control | | $LMNA^{G608G/G608G}$ | |
| sgCtrl | sgOct4 | sgCtrl | sgOct4 |
| 100.8608 | 17.83954 | 129.2898 | 71.11123 |
| 105.1347 | 20.76553 | 130.8675 | 60.76553 |
| 98.5683 | 25.88432 | 102.7656 | 88.67653 |
